# Conjugated fatty acids drive ferroptosis through chaperone-mediated autophagic degradation of GPX4 by targeting mitochondria

**DOI:** 10.1038/s41419-024-07237-w

**Published:** 2024-12-06

**Authors:** Yusuke Hirata, Yuto Yamada, Soma Taguchi, Ryota Kojima, Haruka Masumoto, Shinnosuke Kimura, Takuya Niijima, Takashi Toyama, Ryoji Kise, Emiko Sato, Yasunori Uchida, Junya Ito, Kiyotaka Nakagawa, Tomohiko Taguchi, Asuka Inoue, Yoshiro Saito, Takuya Noguchi, Atsushi Matsuzawa

**Affiliations:** 1 Laboratory of Health Chemistry, Graduate School of Pharmaceutical Sciences, Tohoku University, Sendai, Japan; 2 Laboratory of Molecular Biology and Metabolism, Graduate School of Pharmaceutical Sciences, Tohoku University, Sendai, Japan; 3 Laboratory of Molecular and Cellular Biochemistry, Graduate School of Pharmaceutical Sciences, Tohoku University, Sendai, Japan; 4 Division of Clinical Pharmacology and Therapeutics, Graduate School of Pharmaceutical Sciences, Tohoku University, Sendai, Japan; 5 Laboratory of Organelle Pathophysiology, Department of Integrative Life Sciences, Graduate School of Life Sciences, Tohoku University, Sendai, Japan; 6 Laboratory of Food Function Analysis, Graduate School of Agricultural Sciences, Tohoku University, Sendai, Japan; 7 Graduate School of Pharmaceutical Sciences, Kyoto University, Kyoto, Japan

**Keywords:** Cell death, Drug development

## Abstract

Conjugated fatty acids (CFAs) have been known for their anti-tumor activity. However, the mechanism of action remains unclear. Here, we identify CFAs as inducers of glutathione peroxidase 4 (GPX4) degradation through chaperone-mediated autophagy (CMA). CFAs, such as (10*E*,12*Z*)-octadecadienoic acid and α-eleostearic acid (ESA), induced GPX4 degradation, generation of mitochondrial reactive oxygen species (ROS) and lipid peroxides, and ultimately ferroptosis in cancer cell lines, including HT1080 and A549 cells, which were suppressed by either pharmacological blockade of CMA or genetic deletion of LAMP2A, a crucial molecule for CMA. Mitochondrial ROS were sufficient and necessary for CMA-dependent GPX4 degradation. Oral administration of an ESA-rich oil attenuated xenograft tumor growth of wild-type, but not that of *LAMP2A*-deficient HT1080 cells, accompanied by increased lipid peroxidation, GPX4 degradation and cell death. Our study establishes mitochondria as the key target of CFAs to trigger lipid peroxidation and GPX4 degradation, providing insight into ferroptosis-based cancer therapy.

## Introduction

Ferroptosis is a caspase-independent form of regulated cell death induced by iron- and reactive oxygen species (ROS)-dependent lipid peroxidation [1]. Lipid peroxides, hereafter called lipid ROS, are constitutively generated through the oxidation of polyunsaturated acyl chains of membrane lipids, whose accumulation triggers ferroptosis [1]. Hence, cells are equipped with various antioxidant systems, and the principal enzyme responsible for detoxifying lipid ROS is glutathione peroxidase 4 (GPX4) [2]. GPX4 plays a central role in reducing toxic phospholipid hydroperoxides into non-toxic phospholipid alcohols, at the expense of glutathione (GSH), thereby preventing the accumulation of lipid ROS and, ultimately, ferroptosis. Ferroptosis can be triggered either directly by inhibiting GPX4 or indirectly by reducing GSH levels, underscoring the significance of GPX4 in detoxifying lipid peroxides and cell survival [3].

Ferroptosis was originally discovered by a small molecule screen to identify a potential anti-cancer drug that could specifically induce cell death in rat sarcoma virus (RAS) mutant cancer cells, which led to the identification of various ferroptosis inducers, including RAS-selective lethal 1 (RSL1: Erastin) and RSL3 [1, 2]; Erastin depletes intracellular GSH by blocking cystine incorporation via SLC7A11, while RSL3 directly binds to GPX4 and inhibits its activity. Since the discovery of ferroptosis, significant progress has been made in understanding the roles of ferroptosis in cancer biology and therapy [4]. Ferroptosis has been demonstrated to participate in the functionality of various tumor suppressors, such as p53 and BRCA1-associated protein 1 (BAP1), suggesting that ferroptosis works as a natural impediment to cancer development [5, 6]. Avoidance of ferroptosis via oncogenic signals contributes to tumor initiation, progression, metastasis, and resistance to therapy [7–9]. On the other hand, cancer cells generally have characteristic metabolisms with a high load of reactive oxygen species (ROS), and there are specific mutations that render them inherently susceptible to ferroptosis, conferring the vulnerability for potential therapeutic targets.

Conjugated fatty acids (CFAs) refer to a specific type of polyunsaturated fatty acids characterized by the presence of at least one pair of conjugated double bonds [10, 11]. Due to the difference in the positions of these double bonds, there are various positional and geometric isomers of CFAs. CFAs produced in nature normally include at least one *trans*-double bond and are thus categorized as *trans*-fatty acids (TFAs), falling into two categories: conjugated linoleic acids (CLAs), including rumenic acid (RA; C18:2 *cis*-9, *trans*-11) and its isomer (10*E*,12*Z*)-octadecadienoic acid (C18:2 *trans*-10, *cis*-12, hereafter called 10-CLA), and conjugated linolenic acids (CLNAs), including α-eleostearic acid (ESA; C18:3 *cis*-9, *trans*-11, *trans*-13) and punicic acid (PA; C18:3 *cis*-9, *trans*-11, *cis*-13) [10, 11] (Fig. S1). CLAs are present in various foods, with dairy products and certain meats being the primary sources, while CLNAs are abundant in several dietary oils produced from plants and seeds, including tung, karela, and pomegranate [10, 11]. Whereas industrially produced TFAs, such as elaidic acid (EA, C18:1 *trans*-9), have pro-inflammatory [12–14] and pro-apoptotic activities [15, 16], and are associated with various disorders including cardiovascular and neurodegenerative diseases [17], CLA/CLNAs have been demonstrated to possess various beneficial effects, including anti-tumor, anti-obesity, anti-atherogenic, and anti-diabetic activities [10, 11]. Among these, anti-tumor activity has been most extensively studied; treatment with CLA/CLNAs is specifically cytotoxic to certain types of cancer cells, which has been considered apoptotic cell death, but not to normal cells [10, 11]. However, the precise mechanism of their anti-tumor activity has remained elusive.

A recent report revealed that CLNAs are potent inducers of ferroptosis in triple-negative breast cancer (TNBC) cells [18]. CLNAs, such as ESA, were shown to be incorporated into neutral lipids including triacylglycerol (TAG) present in lipid droplets in a manner dependent on acyl-CoA synthetase long-chain family member 1 (ACSL1) and diacylglycerol acyltransferase 1/2 (DGAT1/2), thereby possibly triggering lipid peroxidation and ferroptosis due to the particularly high susceptibility of CLNAs to oxidation [19]. In the present study, we sought to determine if this mechanism of action of CLNAs is shared with CLAs and is also observed in other types of cells than TNBCs. We report that both CLAs and CLNAs induce ferroptosis through GPX4 degradation via chaperone-mediated autophagy (CMA) by targeting mitochondria in a human sarcoma cell line, HT1080, and a human lung carcinoma cell line, A549. In these cell types, CLA/CLNAs, such as 10-CLA and ESA, trigger ferroptosis in a DGAT1/2-independent manner, but in a manner dependent on ACSL1 that is localized to mitochondria as well as lipid droplets [20], where they promote mitochondrial ROS/lipid ROS generation, leading to CMA-dependent GPX4 degradation. In a tumor xenograft mouse model, oral administration of tung oil, rich in ESA, diminished tumor growth of wild-type (WT) HT1080 cells, but not that of LAMP2A knockout (KO) HT1080 cells, in which CMA was blocked. Our study provides insight into the mechanism of anti-tumor activity of CFAs, and in particular, evidence of mitochondrial ROS/lipid ROS as a driver of CMA leading to GPX4 degradation and, ultimately, ferroptosis.

## Results

### CLA/CLNAs with a *trans* double bond are ferroptosis inducers

**Fig. 1:**
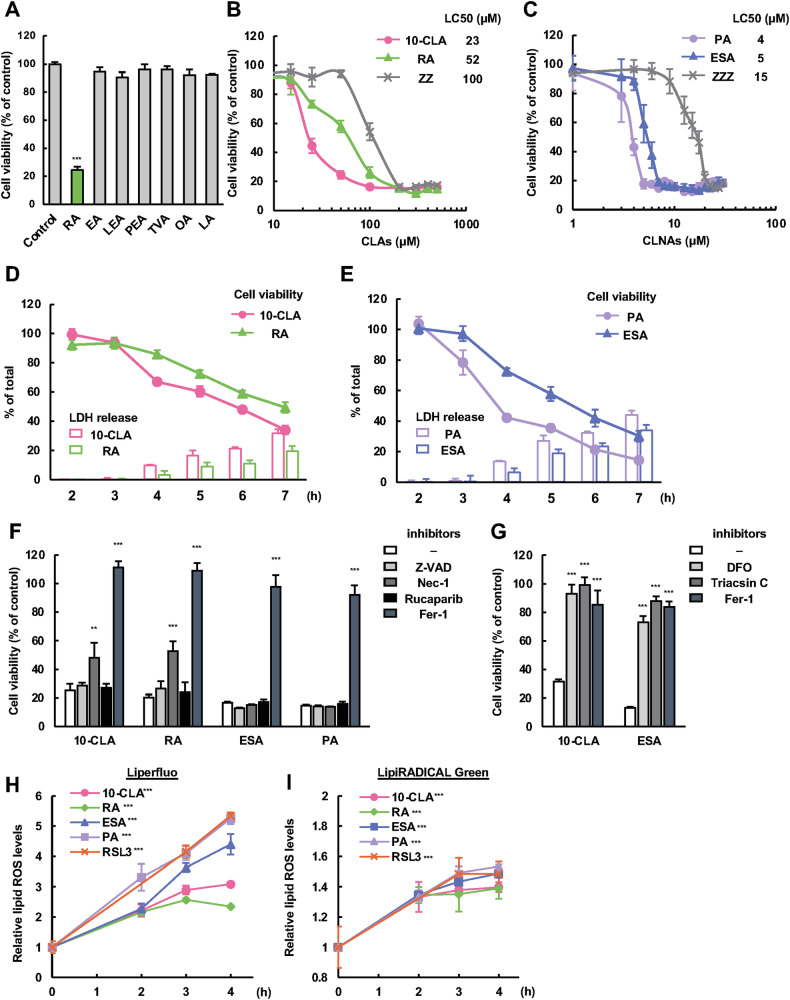
CLA/CLNAs with *trans* double bonds are inducers of ferroptosis. **A** HT1080 cells were treated with RA (C18:2 *cis*-9, *trans*-11), EA (C18:1 *trans*-9), LEA (C18:2 *trans*-9, *trans*-12), PEA (C16:1 *trans*-11), TVA (C18:1 *trans*-11), OA (C18:1 *cis*-9), or LA (C18:2 *trans*-9, *trans*-12) at 200 μM for 24 h, and assayed for cell viability. Data shown are the mean ± SD (*n* = 3). **B**, **C** HT1080 cells were treated with CLAs (10*E*,12*Z* (10-CLA), 9*Z*,11*E* (RA), 9*Z*,11*Z* (ZZ)) (**B**) and CLNAs (9*Z*,11*E*,13*Z* (PA), 9*Z*,11*E*,13*E* (ESA), 9*Z*,11*Z*,13*Z* (ZZZ)) (**C**) at the indicated concentrations for 24 h, and assayed for cell viability. Data shown are the mean ± SD (*n* = 3). **D**, **E** HT1080 cells were treated with either 10-CLA, RA (200 μM) (**D**) PA, or ESA (20 μM) (**E**) for indicated time and assayed for cell viability (lines) and LDH release (bars). Data shown are the mean ± SD (*n* = 3). **F**, **G** HT1080 cells were pretreated with either a pan-caspase inhibitor z-VAD-fmk (20 µM), Nec-1 (30 µM), Rucaparib (1 µM), Fer-1 (5 µM), DFO (100 µM), or Triacsin C (5 µM), for 0.5 h, treated with either 10-CLA, RA (200 µM), ESA, PA (20 µM) for 24 h, and assayed for cell viability. Data shown are the mean ± SD (*n* = 3). **H**, **I** HT1080 cells treated with either 10-CLA, RA (200 µM), ESA, PA (20 µM), or RSL3 (4 µM) for indicated time and lipid ROS levels were measured with Liperfluo (**H**) and LipiRADICAL Green (**I**). Data shown are the mean ± SD (*n* = 3).

RA and 10-CLA, major CLAs in dairy products and meats, have been used as supplements for their various beneficial effects on human health, including anti-obesity and anti-cancer effects, and are considered to be harmless to normal cells and even certain types of cancer cells [21]. Indeed, we previously showed that RA is not cytotoxic to at least multiple cell lines, including mouse macrophage-like cell line RAW264.7, mouse microglial cell line BV2 cells, and human osteosarcoma cell line U2OS [14, 22]. However, when HT1080 cells were treated with various food-derived fatty acids, including five major TFAs, including RA, EA, linoelaidic acid (LEA), palmitelaidic acid (PEA), and *trans*-vaccenic acid (TVA), and two major unsaturated fatty acids, namely oleic acid (OA) and linoleic acid (LA), harboring one or two carbon-carbon double bonds, we observed cytotoxicity only with RA (Fig. 1A). Accumulating evidence has shown anti-cancer activities of CLAs in diverse types of cancer cells, and CLNAs have been demonstrated to kill cancer cells much more efficiently than CLAs [10, 11]. In line with this, dose-response analysis showed more potent cytotoxicity with PA and ESA (5–10 times lower LC50) compared to RA and 10-CLA in HT1080 (Fig. 1B, C) and A549 cells (Fig. S2A, B). Intriguingly, all-*cis* CFAs, including 9Z, 11Z-CLA (*cis* isomer of RA and 10-CLA) and 9Z, 11Z, 13Z-CLNA (*cis* isomer of PA and ESA) (Fig. S1), showed the lowest cytotoxicity among their geometrical isomers (Fig. 1B, C), suggestive of a significance of a *trans* double bond in the anti-cancer activity of CFAs. We next examined the time course of cell death. When treated with CLAs (RA and 10-CLA) at 200 µM, a significant decrease in cell viability along with an increase in LDH release was observed after 4 h (Fig. 1D). Similar time course of changes was observed in the case of CLNAs (PA and ESA) when treated at 20 µM (Fig. 1E). To determine the type of cell death induced by CFAs, various cell death inhibitors were used: Z-VAD (apoptosis inhibitor), Nec-1 (necroptosis inhibitor), Rucaparib (parthanatos inhibitor) and Fer-1 (ferroptosis inhibitor). We found that only Fer-1 completely reversed cell death induced by either RA, 10-CLA (50–400 µM), PA, or ESA (5–40 µM) (Fig. 1F, S2C). Although Nec-1 partially recovered cell death when treated with RA or 10-CLA (Fig. 1F) due to its off-target effect [23, 24], a more specific inhibitor for necroptosis, Nec-1s (7-Cl-O-Nec-1) [25], did not recover it in agreement with a previous report [26] (Fig. S2D). These results indicate that CFAs trigger cell death via ferroptosis. Consistently, cell death induced by 10-CLA and ESA was suppressed by other commonly used ferroptosis inhibitors, including an iron chelator deferoxamine (DFO) and an inhibitor for ACSLs, Triacsin C (Fig. 1G, S2E). Furthermore, the level of lipid peroxidation detected by Liperfluo [27] or LipiRADICAL green [28] was significantly elevated upon treatment with CLAs (RA and 10-CLA) at 200 µM and CLNAs (PA and ESA) at 20 µM (Fig. 1H, I, S3A, B) in a time-dependent manner similarly as when treated with RSL3 at 4 µM (Fig. 1H, I, S3C–E). Note that the sensitivity of cells to RSL3 was reduced by nearly 10-fold in the presence of bovine serum albumin (BSA) (Fig. S3C), which was used to conjugate with fatty acids throughout this study, in line with the previous reports [29, 30]; nonetheless, RSL3, a direct inhibitor of GPX4 (LC50: 1.5 µM) (Fig. S3C), is clearly more potent in inducing ferroptosis than CFAs (LC50: 10-CLA, 23 µM; ESA, 5 µM). These results collectively indicate that CFAs, particularly with a *trans* double bond, are inducers of ferroptosis.

### DGAT1/2 are not involved in CLA/CLNA-induced ferroptosis in HT1080 and A549 cells

**Fig. 2:**
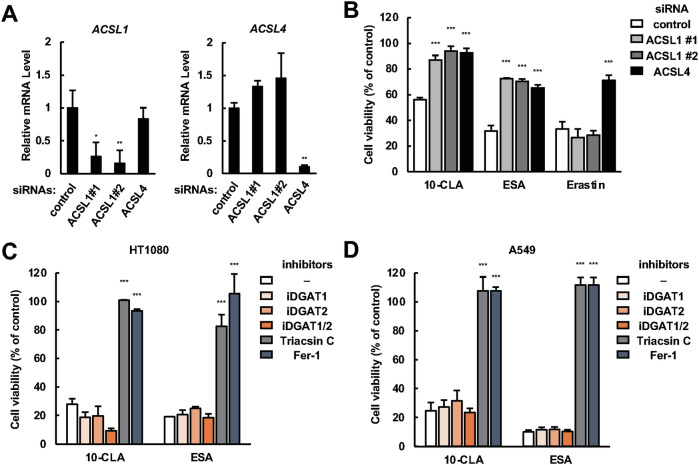
CLA/CLNAs trigger ferroptosis independently of DGAT1/2 in HT1080 and A549 cells. **A** Relative mRNA levels of *ACSL1*, *ACSL4* in control- or *ACSL1/4*-knockdown HT1080 cells are shown as mean ± SD (*n* = 3). **B** Cell viability of control- or *ACSL1/4*-knockdown HT1080 cells treated with either 10-CLA (200 μM), ESA (10 μM) or Erastin (20 μM) for 24 h. Data shown are the mean ± SD (*n* = 3). **C**, **D** HT1080 (**C**) and A549 (**D**) cells were pretreated with either iDGAT1/2 (10 µM), Triacsin C (5 µM), or Fer-1 (5 µM) for 0.5 h, treated with 10-CLA (100 μM: HT1080, 200 µM: A549) or ESA (10 μM: HT1080, 20 µM: A549) for 24 h, assayed for cell viability. Data shown are the mean ± SD (*n* = 3).

A previous study proposed that ACSL1, but not ACSL4, drives DGAT1/2-dependent incorporation of ESA into TAGs localized in lipid droplets, thereby triggering lipid peroxidation in lipid droplets, and, ultimately, ferroptotic cell death [18]. In accordance with this, *ACSL1* knockdown clearly suppressed cell death induced by 10-CLA and ESA, but not that induced by Erastin requiring ACSL4, instead of ACSL1, for inducing ferroptosis [31], in HT1080 cells (Fig. 2A, B). On the other hand, *ACSL4* knockdown reversed both 10-CLA/ESA- and Erastin-induced cell death (Fig. 2B), contrary to the previous report in which *ACSL4* knockout did not affect ESA-induced cell death in Pfa1 (mouse embryonic fibroblast) cells [18]. Moreover, co-treatment of inhibitors for DGAT1 and/or DGAT2 did not reverse cell death upon 10-CLA or ESA treatment in HT1080 and A549 cells (Fig. 2C, D), while partially but significantly reversed it in ESA-treated MDA-MB-468 cells (Fig. S4), consistent with the previous report [18]. Altogether, these results suggest that, although lipid droplets likely contribute to ESA-induced cell death in a DGAT1/2-dependent manner in certain cell types such as TNBCs, there might be other mechanisms that are involved in CLA/CLNA-induced ferroptosis.

### CLA/CLNAs promote lysosomal degradation of GPX4

**Fig. 3:**
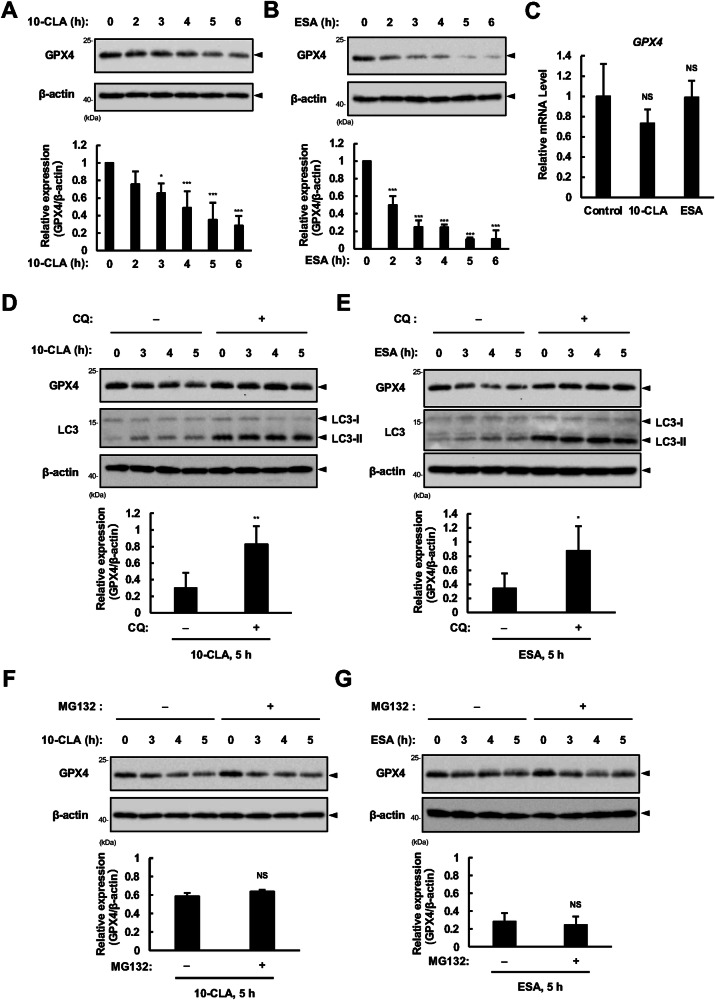
CLA/CLNAs promote lysosomal degradation of GPX4. **A**, **B** Immunoblot of lysates from HT1080 cells treated with 10-CLA (200 µM) (**A**) or ESA (20 µM) (**B**) for indicated time using antibodies against GPX4 and β-actin. Images are cropped for clarity; full-length blots are presented in Supplementary Fig. 13A, B. **C** HT1080 cells were treated with 10-CLA (200 µM) or ESA (20 µM) for 4 h, and relative mRNA level of *GPX4* was measured by qRT-PCR analysis and shown as mean ± SD (*n* = 3). **D**–**G** Immunoblot of lysates from HT1080 cells pretreated with chloroquine (CQ: 20 µM) (**D**, **E**) or MG132 (5 µM) (**F**, **G**) for 0.5 h, and treated with 10-CLA (200 µM) (**D**, **F**) or ESA (20 µM) (**E**, **G**) for indicated time, using antibodies against the indicated proteins. Images are cropped for clarity; full-length blots are presented in Supplementary Fig. 13C–F.

The most critical suppressor of ferroptosis is GPX4 which constitutively converts toxic lipid peroxides to non-toxic lipid alcohols at the expense of GSH; ferroptosis is triggered either directly by inactivating GPX4 or indirectly by GSH depletion [2]. To explore the mechanism of action of CLA/CLNAs, we monitored changes in GPX4 expressions and GSH levels upon 10-CLA/ESA treatment. We found that in response to 10-CLA or ESA, GPX4 protein levels dropped down in a time-dependent manner (Fig. 3A, B) correlating with the elevation of lipid peroxidation levels (Fig. 1H, I), whereas GPX4 mRNA levels were not altered (Fig. 3C). We further checked the changes in protein expression levels of other key molecules in ferroptosis, such as ACSL1/4, BRCA1-associated protein 1 (BAP1) [6], and glutathione synthetase (GSS) [32]; however, none of them were significantly changed (Fig. S5A, B). Also, GSH levels were unchanged after 5 h of CFA treatment (Fig. S5C). Thus, we hereafter focused on the changes in the expression levels of GPX4 protein. The decrease in GPX4 protein level was reversed in the presence of either Fer-1 or DFO, indicating the involvement of lipid peroxidation by 10-CLA/ESA (Fig. S5D, E). Moreover, no significant effect of 10-CLA/ESA on intracellular Fe^2+^ levels was observed (Fig. S6A, B). Together, these data suggest that 10-CLA/ESA specifically facilitate GPX4 protein degradation in a manner dependent on lipid peroxidation. There are two major intracellular protein degradation systems: the autophagy-lysosome degradation system and the ubiquitin-proteasome system [33]. To determine which system is responsible for GPX4 degradation, we utilized specific inhibitors for these degradation systems, an autophagy-lysosome inhibitor, chloroquine (CQ), and a proteasome inhibitor, MG132. As shown in Fig. 3D–G, CQ, but not MG132, substantially prevented downregulation of GPX4 expression upon 10-CLA/ESA treatment, suggesting a crucial role of the autophagy-lysosome degradation system in GPX4 degradation induced by CLA/CLNAs.

### CMA-dependent degradation of GPX4 drives ferroptosis induced by CLA/CLNAs

**Fig. 4:**
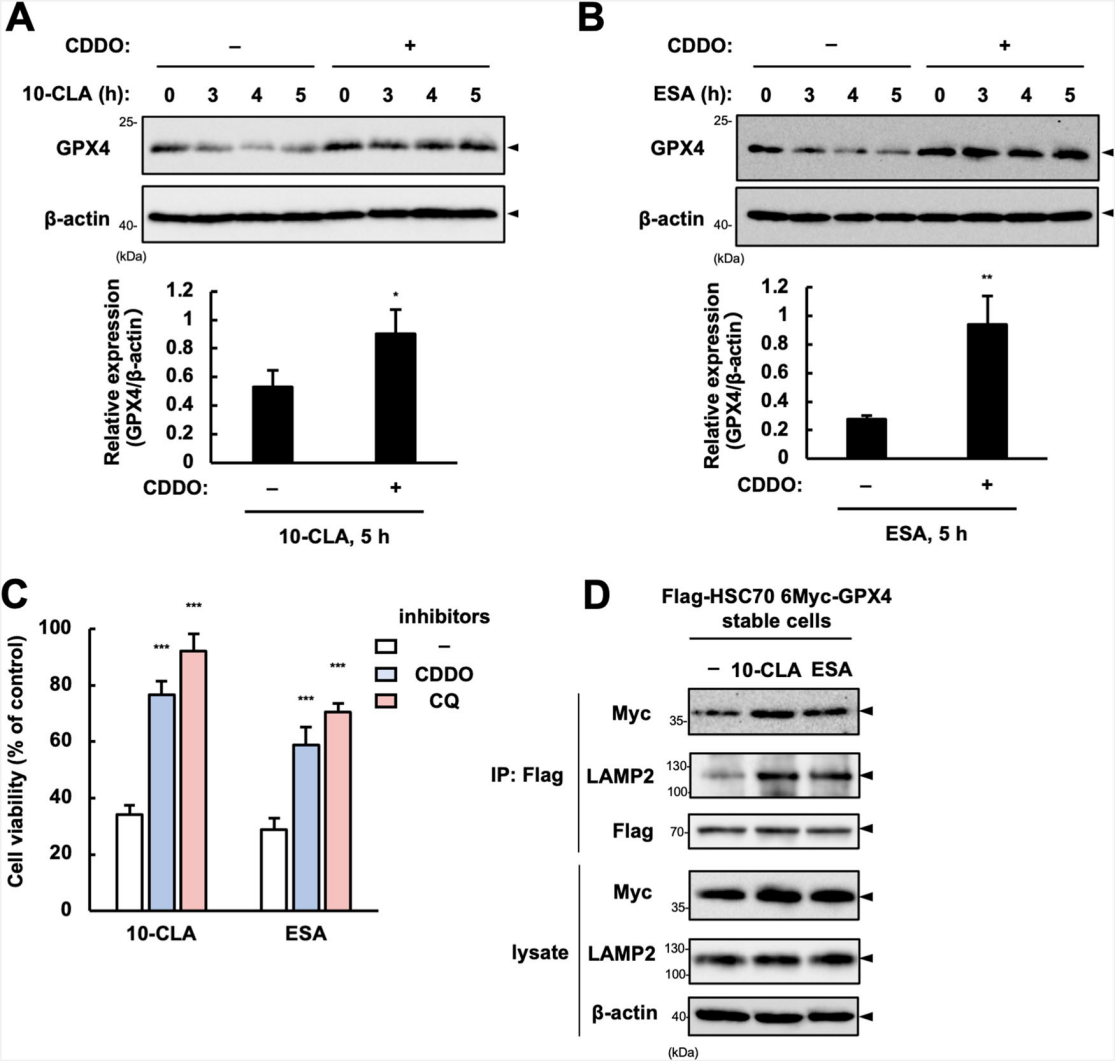
CMA-dependent GPX4 degradation drives ferroptosis in response to CLA/CLNAs. **A**, **B** Immunoblot of lysates from HT1080 cells pretreated with CDDO (5 µM) for 0.5 h and treated with 10-CLA (200 µM) or ESA (20 µM) for indicated time, using antibodies against GPX4 and β-actin. Images are cropped for clarity; full-length blots are presented in Supplementary Fig. 13G, H. **C** HT1080 cells were pretreated with CDDO (5 µM) or CQ (20 µM) for 0.5 h, treated with 10-CLA (100 µM) or ESA (10 µM) for 24 h, and assayed for cell viability. Data shown are the mean ± SD (*n* = 3). **D** HEK293A cells stably expressing Flag-HSC70 and 6Myc-GPX4 were treated with 10-CLA (200 µM) or ESA (200 µM) for 5 h, in the presence or absence of CQ (20 µM) and CDDO (5 µM). Lysates were immunoprecipitated with anti-Flag antibody and subjected to immunoblot analysis using antibodies against the indicated proteins. Images are cropped for clarity; full-length blots are presented in Supplementary Fig. 13I.

**Fig. 5:**
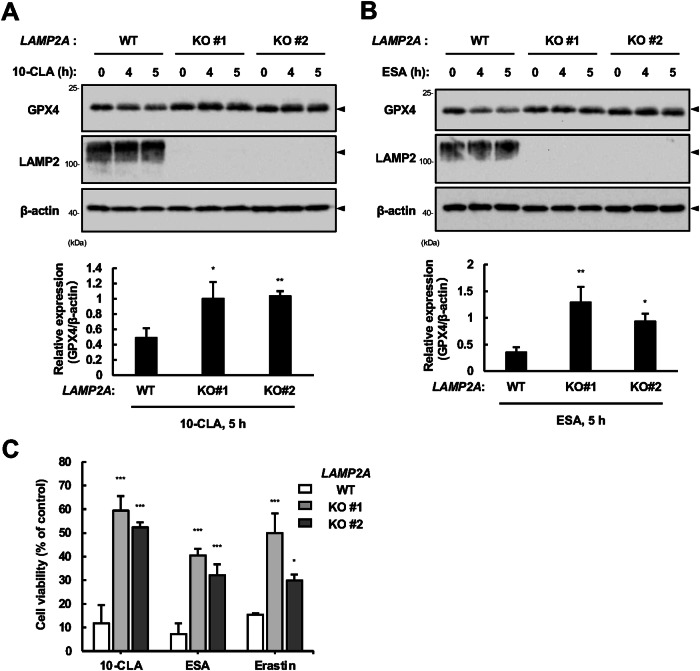
CLA/CLNAs promote GPX4 degradation in a LAMP2A-dependent manner. **A**, **B** Immunoblot of lysates from WT and *LAMP2A* KO HT1080 cells pretreated with CDDO (5 µM) for 0.5 h and treated with 10-CLA (200 µM) or ESA (20 µM) for indicated time, using antibodies against the indicated proteins. Images are cropped for clarity; full-length blots are presented in Supplementary Fig. 13J, K. **C** WT and *LAMP2A* KO HT1080 cells were treated with 10-CLA (100 µM) or ESA (10 µM) or Erastin (20 µM) for 24 h, and assayed for cell viability assay. Data shown are the mean ± SD (*n* = 3).

Three types of autophagy have been identified: macroautophagy, microautophagy, and CMA [34]. The former two require autophagosome formation that depends on LC3, particularly LC3-II conjugated with phosphatidylethanolamine and ATG5 [35]. We observed LC3-II accumulation in parallel with the decrease in GPX4 (Fig. 3D, E) and p62 (Fig. S5A, B) protein levels, implicating the involvement of macroautophagy and/or microautophagy in GPX4 degradation. However, silencing ATG5 did not recover the rate of GPX4 decrease and cell death induced by 10-CLA/ESA (Fig. S7), suggesting that GPX4 degradation is independent of ATG5, a critical regulator of macro- and microautophagy. We thus investigated the potential contribution of CMA, in which molecular chaperones, such as heat shock protein 90 (HSP90) and heat shock cognate protein 70 (HSC70), recognize substrate proteins via KFERQ-like motif, and lysosomal-associated membrane protein 2A (LAMP2A) serves as a substrate receptor on the lysosomal membrane to transport substrates to the lysosome, where they are degraded [34]. GPX4 also harbors the KFERQ-like motif and has been shown to be degraded via CMA in response to GSH depletion by Erastin [35]. As expected, we found that 2-cyano-3, 12-dioxooleana-1, 9(11)-dien-28-oate (CDDO), an inhibitor for heat shock protein 90 (HSP90) commonly used to block CMA [35, 36], effectively reversed GPX4 downregulation (Fig. 4A, B) and cell death as well as CQ (Fig. 4C) upon 10-CLA/ESA treatment. Furthermore, in HEK293A stable cell lines expressing both Flag-HSC70 and 6Myc-GPX4 (Fig. S8A, B), co-immunoprecipitation analysis showed that the interaction of Flag-HSC70 with 6Myc-GPX4 and LAMP2 was increased when treated with 10-CLA/ESA (Fig. 4D). Altogether, these results implicate that treatment with CLA/CLNAs promotes CMA-dependent degradation of GPX4. To further verify these results, we established *LAMP2A* knockout (KO) cell lines in HT1080 cells and confirmed that GPX4 protein was far more stable in *LAMP2A* KO cells than WT cells during 10-CLA/ESA treatment (Fig. 5A, B). We also observed that *LAMP2A* KO cells were strongly resistant to cell death induced by 10-CLA/ESA, as well as to Erastin-induced cell death, in line with the previous report [36] (Fig. 5C). These data collectively suggest that GPX4 degradation via CMA drives ferroptosis induced by CLA/CLNAs.

### CLA/CLNAs potentiate CMA-dependent GPX4 degradation through mitochondrial ROS production

**Fig. 6:**
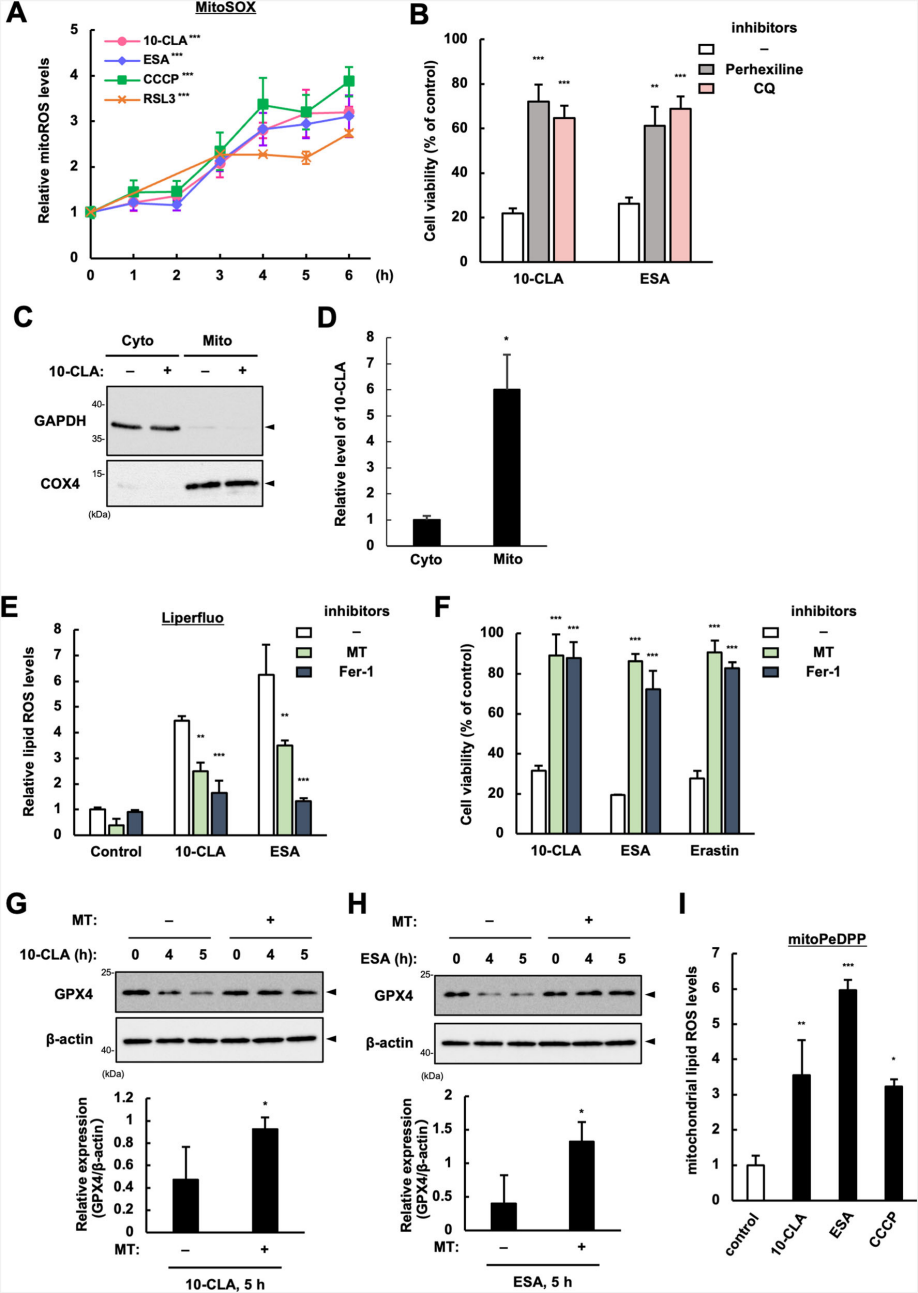
CLA/CLNAs potentiate CMA-dependent GPX4 degradation through mitochondrial ROS production. **A** Mitochondrial ROS level of HT1080 cells treated with either 10-CLA (200 µM) or ESA (20 µM) or CCCP (10 µM) for indicated time, detected with MitoSOX. Data shown are the mean ± SD (*n* = 3). **B** HT1080 cells were pretreated with Perhexiline (7.5 µM) or CQ (20 µM) for 0.5 h, treated with 10-CLA (100 μM) or ESA (10 μM) for 24 h, and assayed for cell viability. Data shown are the mean ± SD (*n* = 3). **C**, **D** HT1080 cells were treated with 10-CLA (200 µM) for 5 h. Lysates were fractionated into cytoplasm and mitochondria, and subjected to immunoblot analysis using antibodies against the indicated proteins (**C**), or to GC-MS analysis (**D**). Relative level of 10-CLA was calculated by dividing the molar amount of 10-CLA by the weight of protein in each fraction and shown as mean ± SEM (*n* = 5). Cyto: cytoplasm, Mito: mitochondria. Images are cropped for clarity; full-length blots are presented in Supplementary Fig. 13L. **E**, **F** HT1080 cells were pretreated with MT (20 µM), Fer-1 (5 µM) for 0.5 h, treated with either 10-CLA (**E**: 200 µM; **F**: 100 µM) or ESA (**E**: 20 µM; **F**: 10 µM) or Erastin (20 µM) for 4 h (**E**) or 24 h (**F**), and assessed their lipid ROS levels using Liperfluo (**E**) and cell viability (**F**). Data shown are the mean ± SD (*n* = 3). **G**, **H** Immunoblot of lysates from HT1080 cells pretreated with MT (20 µM) for 0.5 h and treated with 10-CLA (200 µM) or ESA (20 µM) for indicated time, using antibodies against GPX4 and β-actin. Images are cropped for clarity; full-length blots are presented in Supplementary Fig. 13M, N. **I** Mitochondrial lipid ROS levels of HT1080 cells treated with either 10-CLA (200 µM), ESA (20 µM) or CCCP (10 µM) for 4 h detected with mitoPeDPP. Data shown are the mean ± SD (*n* = 3).

We have shown that 10-CLA/ESA promote ferroptosis by inducing CMA-dependent GPX4 degradation in a manner dependent on ACSL1 (Fig. 2B) but independent of DGAT1/2 (Fig. 2C, D) in HT1080 and A549 cells. Intriguingly, ACSL1 has been shown to be predominantly localized not only at lipid droplets but also at mitochondria [20]. Consistently, immunocytochemical analyses confirmed that a substantial portion of ACSL1 colocalizes with both mitochondrial (translocase of outer mitochondrial membrane 20: TOM20) and lipid droplet markers (adipose differentiation-related protein: ADRP) as well as ACSL4 (Fig. S9). Taking these into consideration, we speculated that in HT1080 and A549 cells, CFAs might be transported primarily to mitochondria, rather than lipid droplets, via ACSL1, thereby facilitating oxidation of mitochondrial molecules, leading to CMA induction. To address this possibility, we first monitored changes in mitochondrial ROS (mitoROS) levels upon 10-CLA/ESA treatment. As shown in Fig. 6A, mitoROS levels were elevated over time, which tracked closely with the change in lipid ROS levels (Fig. 1H, I). As a proof-of-concept experiment, we examined whether inhibiting the delivery of CFAs to mitochondria prevents cell death. Carnitine palmitoyltransferase 1/2 (CPT1/2), mitochondrial transmembrane proteins converting long-chain fatty acyl-CoA to long-chain acylcarnitine, are the rate-limiting enzymes in incorporating fatty acids from cytosol to mitochondria [37]. We showed that CPT1/2 inhibition with Perhexiline strongly suppressed 10-CLA/ESA-induced cell death to the same extent as CQ, implicating a significant role of mitochondrial accumulation of CFAs in ferroptosis (Fig. 6B). Of note, Perhexiline showed no antioxidant activity in vitro, namely 2,2-diphenyl-1-picrylhydrazyl (DPPH) radical scavenging activity, neither did CDDO (Fig. S10A); Perhexiline neither showed an anti-ferroptotic activity to inhibit cell death induced by GPX4 depletion in HT1080 cells (Fig. S10B, C). Moreover, a gas chromatography-mass spectrometry (GC-MS) analysis of cytoplasmic and mitochondrial fractions of HT1080 cells treated with 10-CLA (Fig. 6C) demonstrated that 10-CLA was 6 times more abundant in the mitochondrial fraction than in the cytoplasmic fraction (Fig. 6D). Based on these results, we next tested whether mito-TEMPO (MT), a mitochondria-targeted antioxidant, could alleviate CLA/CLNA-induced ferroptosis. As expected, MT apparently reversed lipid ROS generation (Figs. 6E, S11A, B), and 10-CLA/ESA-induced cell death in HT1080 (Fig. 6F), A549 (Fig. S11C), and MDA-MB-468 cells (Fig. S11D) as well as CQ and CDDO. Of note, MT also prevented Erastin-induced cell death involving CMA-dependent degradation of GPX4, consistent with a previous report (Fig. 6F) [38]. Furthermore, MT effectively inhibited 10-CLA/ESA-induced degradation of GPX4 (Fig. 6G, H). Collectively, these results support our notion that CLA/CLNAs target mitochondria via ACSL1, resulting in CMA induction through the generation of mitoROS and lipid ROS. These observations led to a hypothesis that mitoROS generation drives lipid ROS production, which may in turn induce GPX4 degradation and, ultimately, ferroptosis. Interestingly, a recent report has shown that treatment of HT1080 cells with a typical mitochondrial uncoupler carbonyl cyanide m‐chlorophenylhydrazone, CCCP, commonly used to generate mitoROS, causes lipid ROS production and ferroptotic cell death [39]. We consistently observed that CCCP treatment generated lipid ROS, particularly in mitochondria (Figs. 6I, S12A), detected with a mitochondria-targeted lipid ROS probe (mitoPeDPP) and Liperfluo, along with mitoROS (Fig. 6A), and induced cell death which was significantly suppressed by either Fer-1, MT, or CDDO (Fig. S12B, C). Accordingly, CCCP treatment induced degradation of GPX4, but unexpectedly, reduced GPX4 expression was observed at the same level between WT and *LAMP2A* KO cells (Fig. S12D), while it was significantly prevented in *LAMP2A* KO cells when treated with Erastin in accordance with a previous report [36] (Fig. S12E). Given that CMA does not always require LAMP2A for substrate translocation to lysosomes [40–42], these results implicate that CCCP and Erastin induce CMA-dependent GPX4 degradation in a manner independent and dependent on LAMP2A, respectively. Altogether, these data support our assumption that mitoROS produced by CLA/CLNAs potentiate CMA-dependent GPX4 degradation and lipid peroxidation, resulting in ferroptosis.

### Oral administration of ESA-rich oil inhibits in vivo tumor growth through CMA

**Fig. 7:**
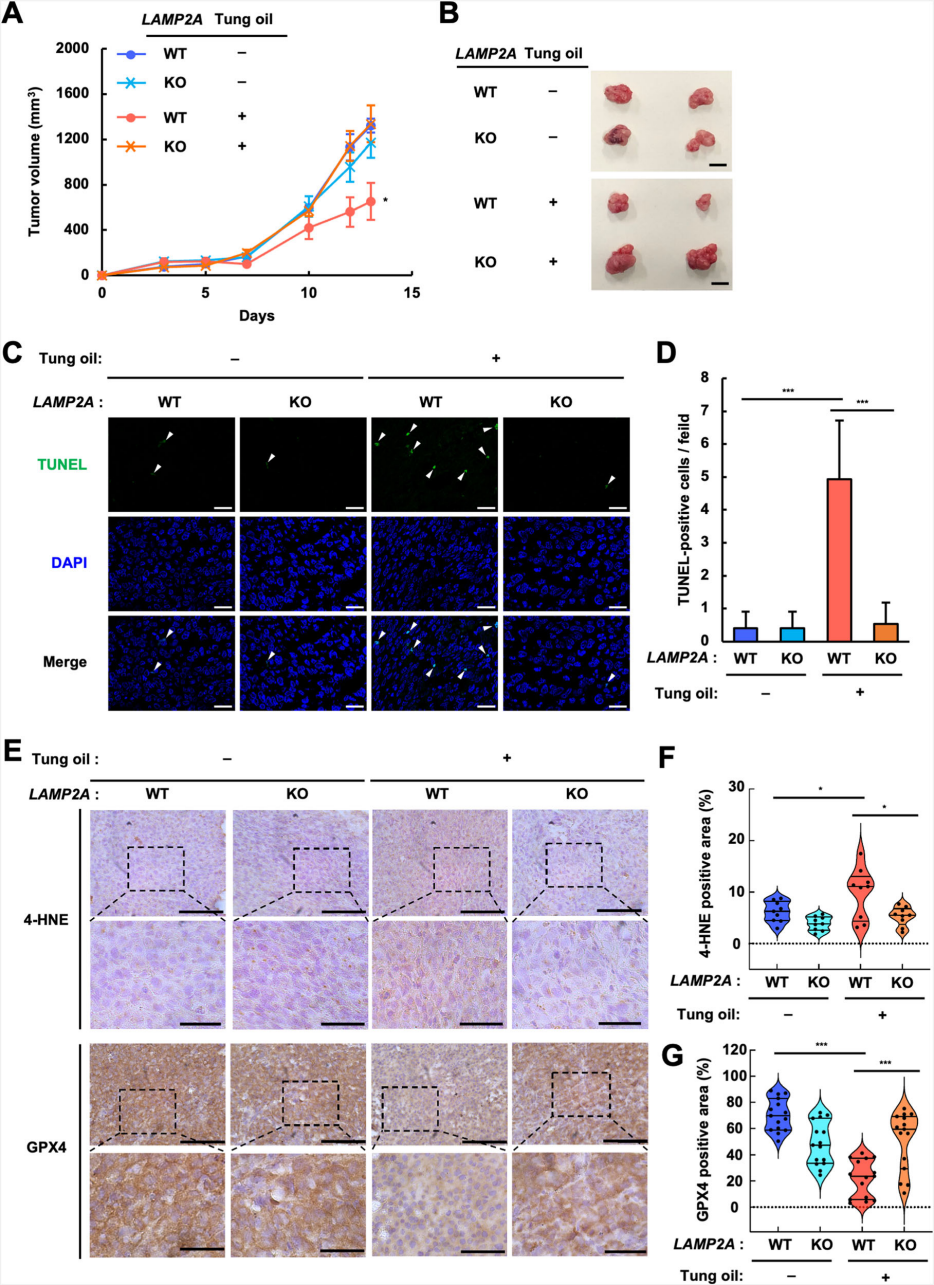
Tung oil rich in ESA attenuates tumor growth by promoting ferroptosis via CMA. **A**, **B** Nude mice were subcutaneously inoculated with WT and *LAMP2A* KO HT1080 cells and administrated orally with either tung oil or safflower oil as control. Tumor volumes were measured three times a week and shown as the mean ± SEM (*n* = 4) (**A**). Representative images of tumors xenografts harvested on day 14 (**B**). ^∗^*p* < 0.05 (vs WT, safflower oil). Bar, 10 mm. **C**, **D** TUNEL staining of paraffin sections of the harvested tumor xenografts counterstained with DAPI (**C**). The arrowheads indicate TUNEL-positive nuclei. White bar, 10 μm. The number of TUNEL-positive nuclei was counted in 4–6 fields for each section from 3 independent tumor xenografts and are shown as an average number of TUNEL-positive cells per field (mean ± SEM, *n* = 3) (**D**). ^∗∗∗^*p* < 0.001 (vs WT, safflower oil). **E**–**G** 4-HNE and GPX4 staining of paraffin sections of the harvested tumor xenografts. The representative images (upper; bar, 100 µm). Inset, magnified images (bottom; bar, 40 µm) (**E**). 4-HNE (**F**) and GPX4 (**G**) positive areas were quantified and shown as percentage of the total areas (violin plot with data points).

We finally tested whether the pro-ferroptotic activity of CFAs depends on CMA in vivo using a tumor xenograft mouse model. Tung oil has a unique fatty acid composition, with ESA accounting for ~80% of total fatty acids [43], and its strong anti-tumor activity has been demonstrated by mounting evidence [18, 44]. Each mouse was inoculated with WT and *LAMP2A* KO HT1080 cells on the left and right side of the back, and treated with either safflower oil, rich in oleic acid (C18:1 *cis*-9) as a control, or tung oil, by oral gavage five times per week. As shown in Fig. 7A, B, tung oil administration significantly suppressed the growth of WT tumors but not that of *LAMP2A* KO tumors, suggesting a contribution of CMA to the anti-tumor activity of tung oil. Consistently, tung oil administration drastically elevated the number of terminal deoxynucleotidyl transferase-mediated dUTP-biotin nick end labeling (TUNEL)-positive cells (dead cells) (Fig. 7C, D) and the level of 4-HNE, a representative lipid peroxidation marker [45], in WT tumors, but not in *LAMP2A* KO tumors (Fig. 7E, F). Conversely, GPX4 protein levels were significantly lowered in WT tumors of mice treated with tung oil (Fig. 7E, G). These data collectively suggest that treatment with CLNAs, such as ESA, diminishes tumor growth in vivo via CMA.

## Discussion

**Fig. 8:**
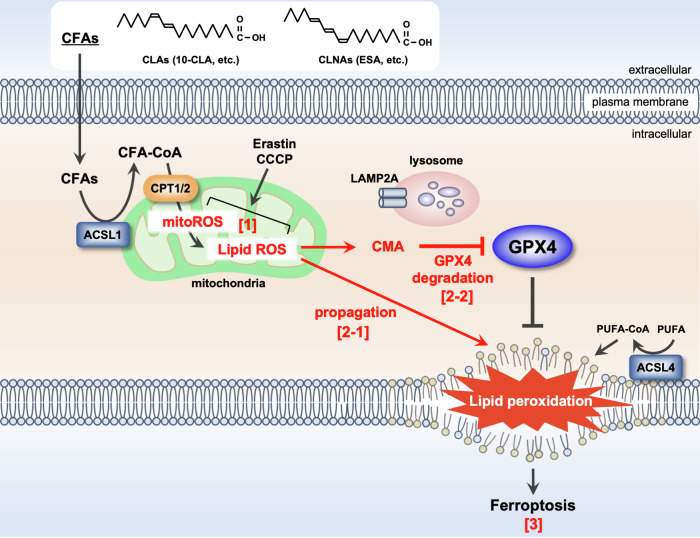
Schematic illustration of the mechanism of pro-ferroptotic actions of CFAs. A proposed model for the molecular mechanism of CFA-induced ferroptosis. CFAs, including 10-CLA and ESA, incorporated into cells accumulate preferentially at the mitochondria via ACSL1 and CPT1/2, primarily responsible for mitochondrial fatty acid transport. Mitochondrial accumulation of CFAs, susceptible to peroxidation due to their conjugated double bonds, initiates the generation of ROS/lipid ROS at mitochondria [1], which in turn triggers both propagation of lipid ROS throughout the cells [2-1], and GPX4 degradation through CMA [2-2], and ultimately, induces ferroptosis [3].

In this study, we established that treatment with CLA/CLNAs, such as 10-CLA and ESA, induces ferroptosis through CMA-dependent degradation of GPX4 by targeting mitochondria, as demonstrated in Fig. 8. A previous study proposed that in TNBCs, such as MDA-MD-468 cells, CLNAs are incorporated preferentially into TAGs that are enriched in lipid droplets, where they are oxidized and produce lipid ROS propagating from lipid droplets to membrane phospholipids, leading to ferroptosis [18]. However, we have shown that inhibitors for DGAT1/2, which are responsible for incorporating CLNAs into TAGs, did not inhibit 10-CLA/ESA-induced ferroptosis in HT1080 and A549 cells (Fig. 2C, D). Instead, either elimination of mitochondrial ROS/lipid ROS with MT or inhibition of mitochondrial transport of fatty acids with Perhexiline clearly suppressed ferroptosis upon 10-CLA/ESA treatment (Fig. 6B, E). We also found that 10-CLA preferentially accumulates in mitochondria (Fig. 6C, D), supporting the significance of mitochondria as the target of CLA/CLNAs. Importantly, MT suppressed ESA-induced ferroptosis much more strongly than iDGAT1/2, even in MDA-MD-468 (Figs. S4B, S11D). These results implicate that mitochondria serve as the major target of CLA/CLNAs not just in HT1080 and A549 cells but also in TNBCs, and the general importance of mitochondria in the pro-ferroptotic activity of these fatty acids among diverse cell types.

CLNAs have been reported to possess much higher potency to produce lipid ROS than their corresponding non-conjugated isomers [19]. The abstraction of a hydrogen atom adjacent to the triple-double bonds occurs 2.5 times faster compared to that of the bis-allylic hydrogen in non-conjugated linolenic acid. Furthermore, peroxyl radicals exhibit an even higher affinity for the triene system of conjugated linolenic acid, whereas they do not significantly add to double bonds in non-conjugated linolenic acid. As a result, the overall rate of free radical oxidation propagation reaches more than 8 times faster for conjugated linolenic acid, explaining their distinct ferroptosis-inducing activity [19]. As for CLAs, their treatment also induced lipid ROS and ferroptotic cell death, although not as potent as CLNAs (Fig. 1). Interestingly, single treatment with polyunsaturated fatty acids, such as arachidonic acids, whose oxidation is known to trigger ferroptosis, do not induce ferroptosis [30], even though their rate of free radical oxidation propagation exceeds that of CLAs [19]. We thus speculate that the specific accumulation of CLAs at mitochondria may play a role in facilitating their oxidation by accelerating CMA-dependent GPX4 degradation.

We observed LC3-II accumulation upon 10-CLA/ESA treatment, indicative of macro- or micro-autophagy induction (Fig. 3D, E). Accumulating evidence has revealed that various types of selective autophagy, such as ferritinophagy, are triggered depending on the stimuli and conditions, and drive cells toward ferroptotic cell death [46]. However, 10-CLA/ESA did not alter the intracellular content of Fe^2+^ or GSH (Figs. S5C, S6). In addition, the knockdown of a key molecule for autophagy, ATG5, did not restore cells from 10-CLA/ESA-induced cell death, but rather exacerbated it (Fig. S7C), presumably due to disruption of mitophagy that is responsible for the removal of mitochondria with accumulated ROS/lipid ROS; indeed, MT, but not CDDO, fully reversed the exacerbated cell death by *ATG5* knockdown (Fig. S7C). On the other hand, both GPX4 degradation and cell death upon 10-CLA/ESA treatment were, not completely but significantly, blocked either pharmacologically with CDDO (Fig. 4A–C) or genetically by *LAMP2A* deficiency (Fig. 5), and almost completely with MT (a mitoROS scavenger) (Figs. 6F–H, S11C, D) or Fer-1 (a lipid ROS scavenger) (Figs. 1F, S2C, S5D, E). Collectively, we propose that the high lipid ROS-producing ability of 10-CLA/ESA is not sufficient, and CMA-dependent degradation of GPX4 is also required for inducing ferroptosis to their full capacity, and more importantly, that mitochondrial ROS/lipid ROS are direct triggers for GPX4 degradation via CMA and subsequent ferroptosis (Fig. 8). As a proof-of-concept experiment, we demonstrated that CDDO could suppress ferroptosis triggered by a typical mitochondrial uncoupler CCCP that induced lipid ROS production and GPX4 degradation (Fig. S12). CMA has been considered to work as a protein quality control by degrading damaged and pathogenic proteins in stress conditions for maintaining cellular homeostasis [34]. Defects in CMA have been associated with various diseases, such as neurodegenerative disorders, heart diseases, and cancers [34]. Intriguingly, compelling evidence has shown that CMA plays an anti-tumorigenic role in non-transformed cells under physiological conditions [34]. Oncogenic proteins, such as murine double minute (MDM2) and translationally controlled tumor protein (TCTP), have been demonstrated to be degraded via CMA [47, 48]. CMA is also protective against malignant transformation by maintaining genome stability through the degradation of checkpoint kinase 1 (Chk1) [49]. As GPX4 has been closely associated with cancer progression and malignancy in various types of cancers [4], GPX4 degradation via CMA might act as a guardian against tumor formation and development; accumulation of mitochondrial ROS/lipid ROS may switch the cell fate from survival to death through specific degradation of GPX4 via CMA. While the contribution of mitochondrial ROS/lipid ROS to CMA-dependent degradation seems clear, much remains to be understood about precisely how mitochondrial ROS/lipid ROS induces CMA-dependent degradation of GPX4, which should be clarified in a future study. It would be also important to know what determines the sensitivity to CLA/CLNAs (e.g., normal cells vs cancer cells, types of cancers, malignancy grade, etc.). Besides, a comprehensive analysis of the metabolism and kinetics of CFAs in the body is warranted as well. Although non-esterified form of CFAs, particularly those conjugated with BSA, has been widely used in previous studies [50, 51], CFAs are mostly taken as phospholipids or triglycerides [52]. Nevertheless, once free CFAs are taken up by cells, most of them are incorporated into phospholipids and triglycerides [18, 53]. Further research is needed on how these esterified CFAs are produced and which forms of CFAs are particularly pro-ferroptotic. Consumptions of CFAs have been known to be safe while attenuating tumor growth in mice and humans [10, 11]. Importantly, epidemiological and clinical studies have shown that the intake of CLA/CLNAs reduced the risk of several types of cancers, such as breast and colorectal cancers [10, 11]. A thorough understanding of the mechanism of action of CLA/CLNA will eventually make these food-derived fatty acids applicable for the prevention and treatment of cancers.

## Materials and methods

### Reagents

All reagents were obtained from commercial suppliers: 1*S*,3*R*-RSL3 (#SML2234), Ferrostatin-1 (#SML0583) (Sigma, Burlington, MA, USA), mito-TEMPO (#sc-221945), Necrostatin-1 (#sc-200142), MG132 (#sc-201270) (Santa Cruz, Dallas, TX, USA), z-VAD-fmk (#3188-v) (Peptide Institute, Osaka, Japan), Necrostatin-1s (7-Cl-O-Nec-1, #S8641), Rucaparib (#S4948) (Selleck, Houston, TX, USA), Liperfluo (#L248), mitoPeDPP (#M466), FerroOrange (#F374) (Dojindo, Kumamoto, Japan), LipiRADICAL Green (#FDV-0042) (Funakoshi, Tokyo, Japan), MitoSOX (#M36008) (Invitrogen, Waltham, MA, USA), Erastin (#17754), Triacsin C (#10007448), PF-04620110 (iDGAT1, #16425), PF-06424439 (iDGAT2, #17680), deferoxamine (DFO, #14595), Perhexiline (#16982), 2-cyano-3, 12-dioxooleana-1, 9(11)-dien-28-oate (CDDO, #81035) (Cayman, Ann Arbor, MI, USA), Chloroquine (#08660-04), Carbonyl Cyanide m-Chlorophenylhydrazone (CCCP, #07253-74) (Nacalai Tesque, Kyoto, Japan).

### Cell culture

HT1080, A549, HEK293A and HEK293T cells obtained from ATCC were cultured in Dulbecco’s Modified Eagle’s medium (#08458-16, Nacalai Tesque) containing 10% (HT1080) or 5% (A549, HEK293A and HEK293T) heat-inactivated fetal bovine serum (FBS) under a 5% CO_2_ atmosphere at 37 °C. MDA-MB-468 cells were cultured in RPMI1640 medium (#30264-56, Nacalai Tesque) containing 10% heat-inactivated fetal bovine serum (FBS) under a 5% CO_2_ atmosphere at 37 °C.

### Preparation and treatment of fatty acids

Fatty acids including oleic acid (OA, #25702-82) (Nacalai Tesque), elaidic acid (EA, #E4637) (Sigma), *trans*-vaccenic acid (TVA, #D-94) (Olbracht Serdary Research Laboratories, Etobicoke, Ontario, Canada), linoleic acid (LA, #90150), linoelaidic acid (LEA, #90160), palmitelaidic acid (PEA, #9001798), (10*E*,12*Z*)-CLA (10-CLA, #90145), (9*Z*,11*E*)-CLA (RA, #90140), (9*Z*,11*Z*)-CLA (#24592), (9*Z*,11*E*,13*E*)-CLNA (ESA, #10008349), (9*Z*,11*E*,13*Z*)-CLNA (PA, #26057) (Cayman), and (9*Z*,11*Z*,13*Z*)-CLNA (ZZZ, #10-1879) (Larodan, Solna, Sweden) were prepared as described previously with minor modifications [14]. Briefly, fatty acids were dissolved in 0.1 N NaOH at 70 °C and then conjugated with fatty acid-free BSA at pH 7.4 (#013-15143) (Wako, Tokyo, Japan) at 55 °C for 10 min to make 5 mM BSA-conjugated fatty acid stock solutions containing 10% BSA. Cells were treated with various concentrations of BSA-conjugated fatty acids by diluting stock solutions in medium with fetal bovine serum (final BSA concentration was set to 1%).

### Cell death assay

Cells were seeded on 96-well plates (Fig. S10C, 2500 cells/well; others, 8000 cells/well). After any stimulation or treatment, lactate dehydrogenase (LDH) assays were performed using the LDH-Cytotoxic Test (#299-50601, Wako) according to the manufacturer’s protocol. The activity level of the LDH released into the culture media is presented as a percentage of the total activity level of LDH, as described previously [54]. The absorbance was read at 570 nm using a microplate reader (iMark, Bio-Rad, Hercules, CA, USA).

### Cell viability assay

Cells were seeded on 96-well plates (5000–10000 cells/well). After any stimulation or treatment, cell viability was determined using the Cell Titer 96 Cell Proliferation Assay (#G5421, Promega), according to the manufacturer’s protocol, as described previously [55]. The absorbance was read at 490 nm using a microplate reader (iMark, Bio-Rad). Data are normalized to control without stimulus. The lethal concentration 50 (LC50) value was calculated using GraphPad Prism.

### qRT-PCR analysis

Total RNA was extracted with Sepasol RNA I Super (#09379-55, Nacalai Tesque) and reverse transcripted into cDNA with High-Capacity cDNA Reverse Transcription Kit (#4368814) (Thermo Fisher Scientific, Waltham, MA, USA). Relative mRNA levels of target genes were determined by real-time quantitative fluorescence PCR (qRT-PCR) using Luna Universal qPCR Master Mix (#M3003E) (New England Biolabs, Ipswich, MA, USA) according to the manufacturer’s protocol, and normalized with those of *GAPDH*. Sequences of primers used in this are:

*GPX4*-forward, 5’-ACCGAAGTAAACTACACTCAG-3’

*GPX4*-reverse, 5’-GGCGAACTCTTTGATCTCTT-3’

*ACSL1*-forward, 5’-GACATTGGAAAATGGTTACCAAATG-3’

*ACSL1*-reverse, 5’-GGCTCACTTCGCATGTAGATA-3’

*ACSL4*-forward, 5’-TCTTCTCCGCTTACACTCTCT-3’

*ACSL4*-reverse, 5’-CTTATAAATTCTATCCATGATTTCCGGA-3’

*GAPDH* -forward, 5’-AACAGCCTCAAGATCATCAGC-3’

*GAPDH* -reverse, 5’-GGATGATGTTCTGGAGAGCC-3’.

### Flow cytometry

For detection of lipid peroxidation, mitochondrial lipid peroxidation, and mitochondrial ROS, HT1080 cells seeded on 12-well plates (120,000 cells/well) were treated with either 1 μM Liperfluo or 2 µM LipiRADICAL green, 0.5 µM mitoPeDPP, and 5 µM MitoSOX, respectively, for 30 min before collection by trypsinization. Fluorescence levels of collected cells were measured by a flow cytometer (CytoFLEX, Beckman Coulter, Brea, CA, USA) with an excitation wavelength of 488 nm and an emission wavelength of 525 nm (Liperfluo, LipiRADICAL green and mitoPeDPP) or 585 nm (MitoSox). Data of 10,000 events per sample were acquired and analyzed by CytoExpert (Beckman Coulter).

### Fe^2+^ fluorescent imaging

HT1080 cells seeded on 96-well glass bottom plates (10,000 cells/well) were treated with 1 μM FerroOrange [56] for 30 min before observation by a fluorescence microscopy (BZ-X800L, Keyence, Osaka, Japan) with an excitation wavelength of 545 nm and an emission wavelength of 605 nm. After subtraction of background fluorescence intensity, mean red fluorescence intensity was calculated for each cell using ImageJ.

### siRNA transfection

HT1080 cells were transfected with 10 nM non-targeting siRNA pool (#D-001206-13, Dharmacon) as control or siRNAs targeting either *ACSL1, ACSL4* or *ATG5* using Lipofectamine RNAiMAX Transfection Reagent (#13778150, Invitrogen), according to the manufacturer’s instructions.

*ACSL1* #1, 5’-CAUAGUGAGCGAUUGUUCA-3’

*ACSL1* #2, 5’-GUGUGAAAGGGCCAAAUGU-3’

*ACSL4*, 5’- GAUCUAGUGAAGUUACAAG-3’

*ATG5* #1, 5’-UCCAACUUGUUUCACGCUAUA-3’

*ATG5* #2, 5’- CUAGGAGAUCUCCUCAAAGAA-3’

### GSH measurement

HT1080 cells were seeded on 12-well plates (120,000 cells/well), and treated with either CFAs or Erastin. Cells were collected in 200 µL 5% (w/v) trichloroacetic acid with 5 mM EDTA, and centrifuged at 3000×*g* for 10 min at 4 °C. After centrifugation, 100 µL of supernatant was mixed with 350 µL 1 M potassium borate buffer (pH 10.5) containing 5 mM EDTA, and 50 µL 0.2% 7-fluorobenzo-2-oxa-1,3-diazole-4-sulfonic acid ammonium (SBD-F) solution, and heated at 60 °C for 30 min. After cooling to room temperature, 50 µL 4 M HCl was added to terminate the reaction. HPLC was performed as described previously [57]. The fluorescence of SBD derivatives was monitored at 516 nm after excitation at 384 nm. Column: ODS-80TM (Tosoh Bioscience, Tokyo, Japan), elution: a mixture of 0.1 M citrate buffer (pH 3.2) and acetonitrile (96:4, v/v), flow rate: 1.0 mL/min. Calibration curves were prepared by fluorescence derivatization of pure GSH solutions by the same method as above, and the retention time of GSH was confirmed in the condition. Intracellular GSH level of each sample was normalized with cell viability.

### Generation of stable cells

A stable cell line expressing Flag-HSC70 and 6Myc-GPX4 was generated by retroviral transduction as follows. cDNAs encoding human HSC70 and GPX4 were obtained by performing PCR and were inserted into pMXs-IP [58] with Flag tag and pMXs-IH [59] with 6Myc tag, respectively. Packaging cell line Phoenix-AMPHO was transfected with pMXs-IH 6Myc-GPX4. After 48 h of culture, the growth medium containing retrovirus was collected. HEK293A cells were incubated with the virus-containing medium with 10 µg/mL polybrene for 48 h, and uninfected cells were eliminated by selection with hygromycin to establish a 6Myc-GPX4 stable cell line. Afterward, Flag-HSC70 was introduced into this stable cell line by the same method using pMX-IP Flag-HSC70 and puromycin as a selection marker to obtain a cell line stably expressing both Flag-HSC70 and 6Myc-GPX4.

### Generation of knockout cell lines

*LAMP2A-* and *GPX4-* knockout cells were generated using the CRISPR/Cas9 system [60, 61]. Guide RNA (gRNA) was designed to target exon 2 of *LAMP2A* gene (5’-AGCTGTGCGGTCTTATGCAT-3’) and exon 2 of *GPX4* gene (5’-CTTGGCGGAAAACTCGTGCA-3’) using CRISPRdirect [62]. gRNA-encoding oligonucleotides were cloned into lentiCRISPRv2 plasmid [63], and the plasmid was transfected with HEK293T cells together with a packaging plasmid psPAX2 and an envelope plasmid pVSV-G. The virus-containing supernatants were collected and used for infecting HT1080 cells, and then infected cells were selected with puromycin and cloned by limiting dilution. To check whether the mutation in *LAMP2A* was introduced in the cloned cells, genomic sequences around the target region were analyzed by PCR-direct sequencing using the following primers.

Forward, 5’-AGCCATGACCTAAGCATCCTA-3’

Reverse, 5’-TTTTGCATAAAGGCAAGTGGC-3’

Also, depletion of the protein expressions of LAMP2A and GPX4 in the cloned cells was verified by immunoblot analysis. As *GPX4* KO cells are lethal without a lipophilic antioxidant, *GPX4* KO cells were maintained in the culture medium with 2.5 µM Fer-1.

### Immunoblot analysis

Cells were lysed in ice-cold lysis buffer containing 20 mM Tris-HCl, pH 7.4, 150 mM NaCl, 1% Triton-X100, 10% Glycerol, and 1% protease inhibitor cocktail (#25955-11, Nacalai tesque). After centrifugation, the cell extracts were resolved by SDS-PAGE and were analyzed as described previously [64]. The blots were developed with ECL (Merck Millipore, Burlington, MA, USA), and detected with ChemiDoc Touch Imaging System (Bio-Rad). The following antibodies were used for immunoblotting: anti-GPX4 (#ab125066) (Abcam, Cambridge, UK), anti-LC3 (#PM036), anti-p62 (#PM045) (MBL, Tokyo, Japan), anti-LAMP2 (#sc-18822), anti-ATG5 (#sc-133158), anti-β-actin (#sc-47778), anti-GSS (#sc-166882) (Santa Cruz), anti-ACSL1 (#13989-1-AP), anti-ACSL4 (#22401-1-AP), and anti-BAP1 (#10398-1-AP) (Proteintech, Rosemont, IL, USA). Band intensity was quantified with Image Lab software (BioRad, version 6.1.0), normalized with that of β-actin, and shown as the mean ± SD (*n* = 3) unless noted otherwise.

### Immunoprecipitation

HEK293A stable cells were seeded at 2 × 10^6^ cells/dish in 10 cm dish. After treatment with fatty acids, cells were harvested, lysed in ice-cold lysis buffer, and centrifuged (15,000 rpm, 15 min, 4 °C). The supernatant was subjected to immunoprecipitation with anti-Flag antibodies (anti-Flag affinity M2 gel, #A2220, Sigma). The immunoprecipitates were subsequently washed with lysis buffer, and subjected to immunoblot analysis.

### Immunocytochemistry

Immunocytochemistry was performed as described previously with minor modifications [65]. HT1080 were treated with or without CFAs for 4 h. Cells were fixed with 3.7% formaldehyde, permeabilized with 50 µg/mL digitonin (#D141, Sigma), blocked with 3% BSA-PBS, and incubated with primary antibodies overnight at 4 °C, followed by incubation with secondary antibodies with DAPI for 1 h at room temperature. The immunostained samples were enclosed with Fluoro-KEEPER Antifade Reagent (#12593-64, Nakalai Tesque), and observed with a Zeiss LSM900 confocal fluorescence microscope. The antibodies used are as follows: rabbit anti-ACSL1 (#13989-1-AP, 1:200), rabbit anti-ACSL4 (#22401-1-AP, 1:200), mouse anti-ADRP (#15294-1-AP, 1:200) (Proteintech), mouse anti-TOM20 (#sc-17764, 1:1000) (Santa Cruz), goat anti-rabbit Alexa-488 (#A-11008, 1:1000), and goat anti-mouse Alexa 555 (#A-21422, 1:1000) (Invitrogen). Quantification of colocalization was performed using ImageJ and shown as a violin plot with data points (*n* = 15; 5 images per sample, 3 samples).

### Preparation of cytoplasmic and mitochondrial fractionations

Fractionation was performed as described previously with minor modifications [15]. HT1080 cells were seeded at 2 × 10^6^ cells/dish in 10 cm dish, and 3 dishes of cells were used for one sample. Cells were washed twice with PBS and scraped with 1 mL of PBS per dish. Cells were pelleted by centrifugation at 1000×*g* for 15 min at 4 °C. The pellet was resuspended in 200 μL of cell homogenization buffer (10 mM Tris-HCl, pH 6.7, 150 mM MgCl_2_, 10 mM KCl) on ice. The dounce homogenizer (loose, #357538, Wheaton) was used to disrupt the cells with 100 up-and-down strokes on ice. To the homogenized cells, 80 μL of cell homogenization buffer containing 0.25 M sucrose was added and mixed by inversion. Nuclei and uncrushed cells were removed as pellets by centrifugation at 1000×*g* for 5 min at 4 °C. The supernatant was centrifuged at 5000×*g* for 10 min at 4 °C, and the resultant supernatant was used for experiments as the cytoplasmic fraction. The pellet was resuspended in 200 μL of ice-cold sucrose/Mg^2+^ buffer (150 mM MgCl_2_, 0.25 M sucrose, 10 mM Tris-HCl, pH 6.7). The dounce homogenizer (tight, #357538, Wheaton) was used to disrupt the cells with 100 up-and-down strokes on ice. The suspension was centrifuged at 5000×*g* for 10 min at 4 °C, and the resultant pellet was suspended in 30 μL of ice-cold lysis buffer and used for experiments as the mitochondrial fraction. The purity of each fraction was determined by immunoblot analysis with antibodies against a mitochondrial marker, COX4 (#11242-1-AP, Proteintech), and a cytoplasmic marker, GAPDH (#01625523, Wako).

### GC-MS analysis

Lipid extraction and GC-MS analysis for 10-CLA were performed as described previously [14]. Lipids were extracted by the Bligh and Dyer method [66]. Isolated lipids were methylated with 1% H_2_SO_4_ in methanol, and the resultant fatty acid methyl esters were extracted with hexane, subjected to GC-MS analysis. GC-MS analysis was performed using GCMS-QP2010 Plus (Shimadzu, Kyoto, Japan) equipped with Zebron ZB-FAME 60 m × 0.25 mm × 0.20 µm (Phenomenex, Torrance, CA, USA). The amounts of 10-CLA in the cytoplasmic and mitochondrial fractions were calculated based on standard curves created from serial dilution of the respective fatty acids, which was normalized with the amount of extracted protein measured by Bradford method using protein assay CBB solution (#29449-15, Nacalai Tesque).

### DPPH assay

Antioxidant activity was assayed using DPPH (#D678, Dojindo) according to the manufacturer’s protocol with minor modifications. Each compound was dissolved in 10 µL ethanol at different concentrations, and mixed with 40 µL of the assay buffer and 50 µL of the DPPH working solution. After incubation at room temperature for 30 min, absorbance at 517 nm was measured using a microplate reader.

### In vivo tumor xenograft model

BALB/cA-*nu*/*nu* male nude mice (6 weeks old) were obtained from CLEA Japan. Both *LAMP2A* WT and KO HT1080 cells were subcutaneously injected into a nude mouse (5,000,000 cells in 0.2 mL PBS/mouse). From day 3, mice were randomly divided into two groups and treated with either safflower oil (Nisshin, Tokyo, Japan) and Tung oil (Sigma) at a dose of 100 µL by oral gavage once every weekday. Tumor size was measured three times a week using a caliper and calculated using the following formula: tumor volume = length × width^2^ / 2. After 14 days, animals were sacrificed, and the xenograft tumors were harvested. Mice were maintained according to the Guidelines for Animal Experimentation of Tohoku University, and all the procedures were approved by the Institutional Animal Care and Use Committee at Tohoku University (approval number: 2022PhA-008). No sample size estimation or blinding was conducted.

### Immunohistochemistry

Harvested xenograft tumors were fixed with 4% paraformaldehyde and paraffin-embedded. Slides sectioned at 5 µm thickness were deparaffinized by xylene and rehydrated through a series of graded alcohol. Heat-mediated antigen retrieval was performed with pH 6 citrate buffer for 10 min at 121 °C. Afterward, the slides were cooled down for 20 min at room temperature. In 4-HNE staining, the sections were treated with 3% H_2_O_2_ for 10 min to block endogenous peroxidase, incubated with 3% BSA-PBS for 1 h for blocking, and incubated with mouse monoclonal anti-4HNE antibody (Abcam) at 1:100 (1 µg/mL) dilution for 1 h at room temperature. The antigen-antibody reactions were detected with goat anti-mouse IgG antibody conjugated with HRP and visualized with 3,3-diaminobenzadine (DAB, #25985-50, Nacalai Tesque) for 10 min. GPX4 staining was performed as described previously with modifications [67]. The procedures up to antigen retrieval were the same as for 4-HNE staining. After antigen retrieval, the sections were treated with 3% H_2_O_2_ for 15 min, incubated in 3% BSA-PBS for 1 h, followed by incubation with anti-GPX4 at 1:100 dilution for 1 h at room temperature. Afterwards, the sections were incubated with a second Ab, biotinylated rabbit anti-rat IgG Ab (#BA400, Funakoshi), at 1:200 dilution for 15 min, followed by incubation with ExtrAvidin-peroxidase (#E2886, Sigma) at 1:50 dilution for 20 min. The sections were incubated with DAB (Nacalai Tesque) to develop a color signal. Sections were counterstained with hematoxylin, dehydrated with a series of ethanol and xylene, and coverslipped before examination by light microscopy. The obtained images were analyzed with ImageJ (3–5 images from 3 tumors), and DAB-positive areas were shown as the percentage of the total areas.

### TUNEL staining

TUNEL staining was performed with In Situ Cell Death Detection Kit, Fluorescein (#11684795910) (Merck Millipore) according to the manufacturer’s instructions, as described previously [65]. Briefly, harvested xenograft tumors were fixed with 4% paraformaldehyde, and embedded in paraffin. 5-μm paraffin sections were TUNEL-labeled, counterstained with Fluoro-KEEPER Antifade Reagent (Non-Hardening Type with DAPI, #12745-74, Nacalai Tesque), and observed with a fluorescence microscope (BZ-X800L, Keyence). Cells with green fluorescence with DAPI signal (namely, nucleus) were defined as TUNEL-positive cells, and the number of TUNEL-positive cells was counted.

### Statistical analysis

All the values are expressed as the mean ± standard deviation (S.D.), and statistical analysis was performed using GraphPad Prism software (Version 9.5.1) (GraphPad Software, Boston, MA, USA). All experiments were repeated at least three independent times. Two groups were compared using two-tailed Student’s *t*-test. Multiple-group comparisons were conducted using the one-way or two-way ANOVA analysis followed by Tukey’s or Dunnett’s test. Significant differences were determined versus control unless noted otherwise, and represented as follows: NS, not significant; *p < 0.05; **p < 0.01; ***p < 0.001.

## Supplementary information

Supplementary Figures

## Data Availability

The datasets used and/or analyzed during the current study are available from the corresponding author upon reasonable request.
