# Supplementary material for: Conjugated fatty acids drive ferroptosis through chaperone-mediated autophagic degradation of GPX4 by targeting mitochondria
Source: Cell Death Dis. 2024 Dec 6;15(12):884. doi: 10.1038/s41419-024-07237-w (PMC11624192; doi:10.1038/s41419-024-07237-w)
Supplement: Supplementary file 1 — Supplementary Figures [file 41419_2024_7237_MOESM1_ESM.pdf]

Supplementary Information

Conjugated fatty acids drive ferroptosis through chaperone-mediated autophagic degradation of GPX4 by targeting mitochondria

Yusuke Hirata, Yuto Yamada, Soma Taguchi, Ryota Kojima, Haruka Masumoto, Shinnosuke Kimura, Takuya Niijima, Takashi Toyama, Ryoji Kise, Emiko Sato, Yasunori Uchida, Junya Ito, Kiyotaka Nakagawa, Tomohiko Taguchi, Asuka Inoue, Yoshiro Saito, Takuya Noguchi, and Atsushi Matsuzawa

Included materials

・**Figure S1-S13**

conjugated linoleic acids (CLAs)

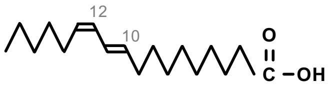

10*E*,12*Z*-CLA
(10-CLA, C18:2 *t10*,*c*12)

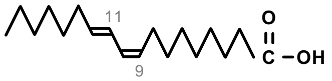

9*Z*,11*E*-CLA
(Rumenic acid [RA], C18:2 *c*9,*t*11)

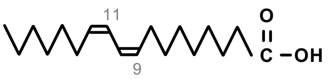

9*Z*,11*Z*-CLA
(C18:2 *c*9,*c*11)

conjugated linolenic acids (CLNAs)

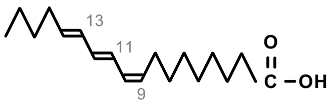

9*Z*,11*E*,13*E*-CLNA
(α-Eleostearic acid [ESA], C18:3 *c*9,*t*11,*t*13)

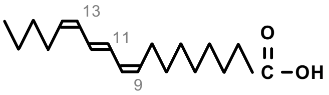

9*Z*,11*E*,13*Z*-CLNA
(Punicic acid [PA], C18:3 *c*9,*t*11,*c*13)

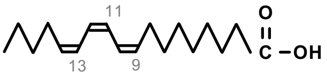

9*Z*,11*Z*,13*Z*-CLNA
(C18:3 *c*9,*c*11,*c*13)

Supplementary Figure 1. Structures of the conjugated fatty acids utilized in this study.
The gray numbers in the figure represent the positions of carbon-carbon double bonds. In parentheses, the following information is shown: common name, abbreviation, the number of carbon atoms, and the number, configuration (*t*, *trans*; *c*, *cis*) and positions of the carbon-carbon double bonds.

Hirata et al., Fig. S1.

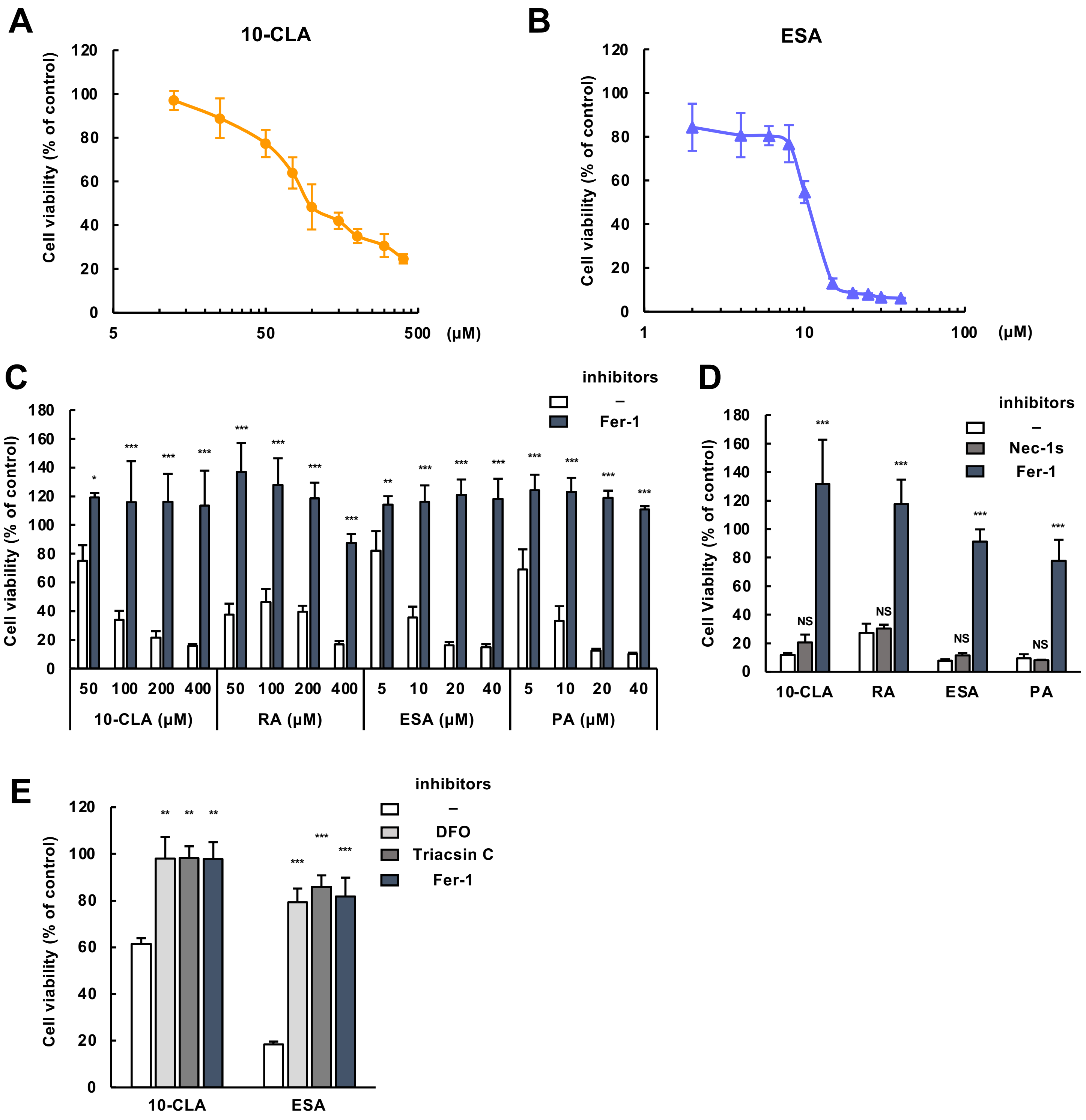

Supplementary Figure 2. CLA/CLNAs induce ferroptosis in HT1080 and A549 cells. Related to Figure 1.

(A, B) Dose-response curve of CFAs for A549 cells. A549 cells were treated with 10-CLA (A) and ESA (B) at the indicated concentrations for 24 h, and assayed for cell viability. Data shown are the mean ± SD (n = 3). LC50: 10-CLA, 78 μM; ESA, 11 μM.

(C, D) HT1080 cells were pretreated with Fer-1 (5 μM) or Nec-1s (30 μM) for 0.5 h, treated with the indicated concentrations of 10-CLA, RA (200 μM in D), ESA, or PA (20 μM in D) for 24 h, and assayed for cell viability. Data shown are the mean ± SD (n = 3). (E) A549 cells were pretreated with either the iron chelator DFO (100 μM), pan-ACSL inhibitor Triacsin C (5 μM), Fer-1 (5 μM) for 0.5 h, treated with 10-CLA (200 μM) or ESA (20 μM) for 24 h, and assayed for cell viability. Data shown are the mean ± SD (n = 3).

Hirata et al., Fig. S2.

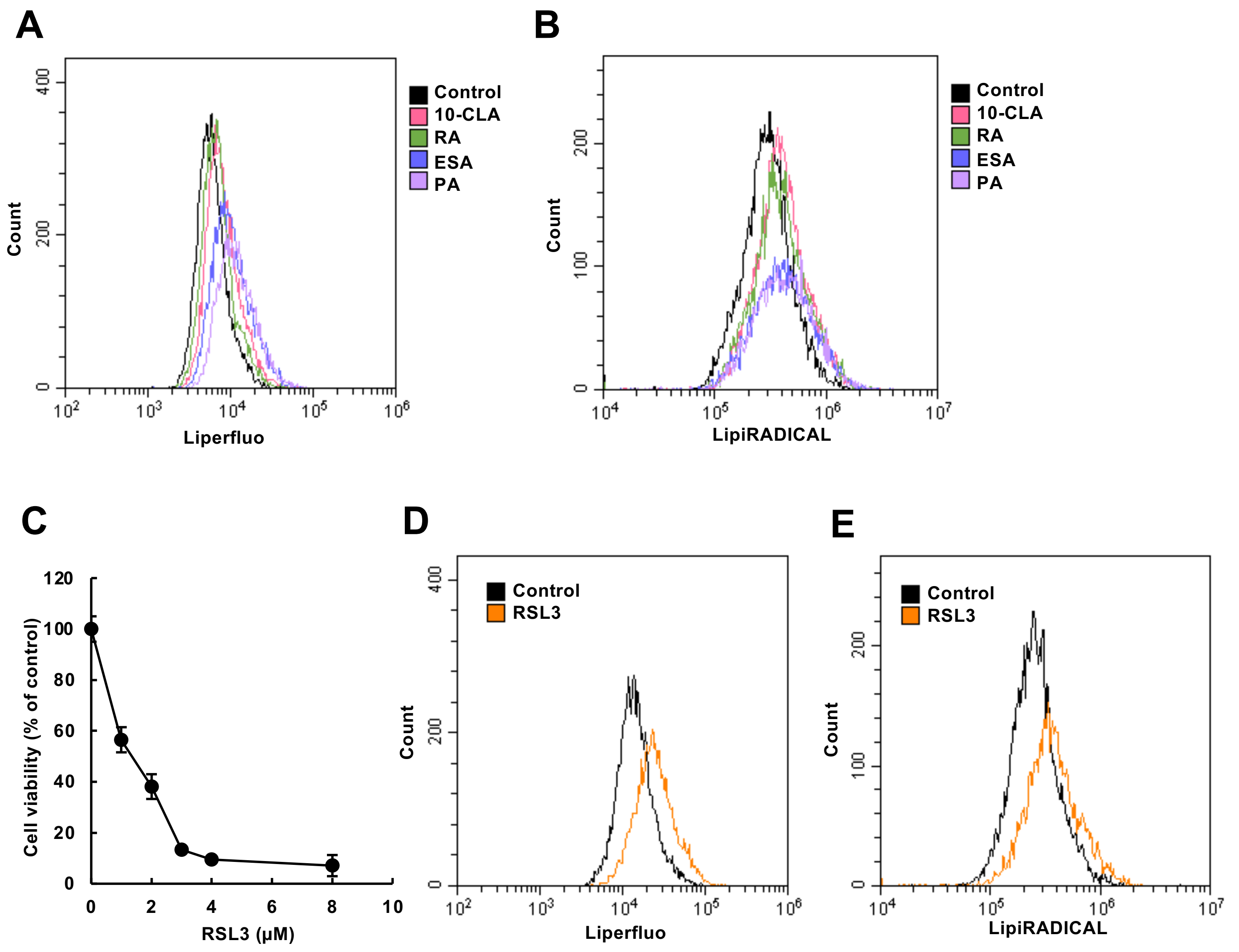

Supplementary Figure 3. CLA/CLNAs induce lipid peroxidation in HT1080 cells. Related to Figure 1.
(A, B) Representative histograms of flow cytometric analysis for HT1080 cells treated with CLA/CLNAs in Fig. 1H (A) and Fig. 1I (B). (C) HT1080 cells were treated with the indicated concentrations of RSL3 in the presence of BSA for 24 h, and assayed for cell viability. Data shown are the mean ± SD (n = 3) (C). (D, E) Representative histograms of flow cytometric analysis for HT1080 cells treated with RSL3 (4 μM) in Fig. 1H (D) and Fig. 1I (E).

Hirata et al., Fig. S3.

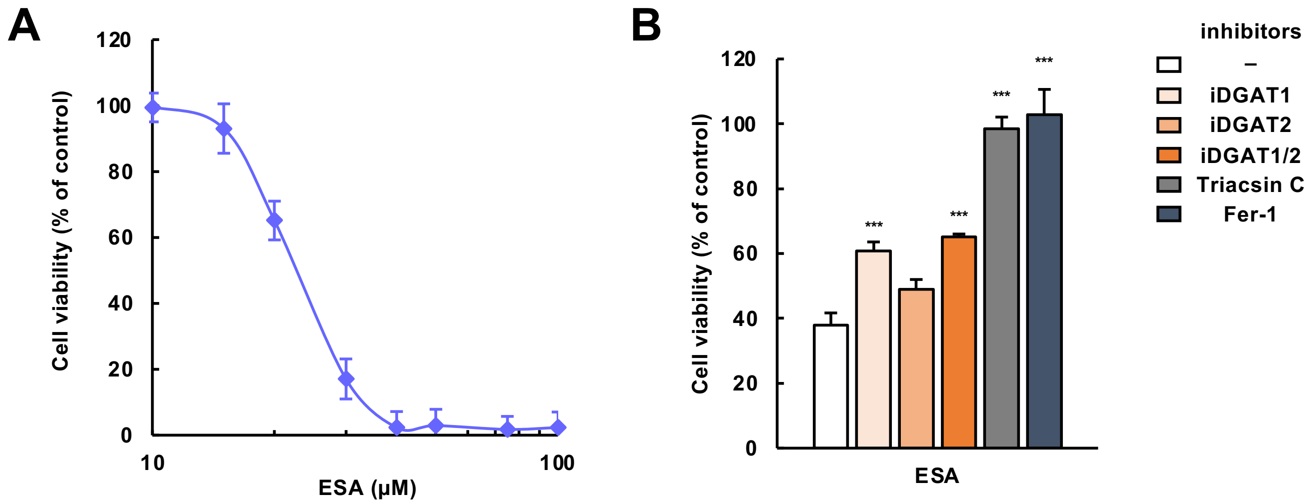

Supplementary Figure 4. ESA induces ferroptosis in MDA-MB-468 cells in a manner partially dependent on DGAT1/2. Related to Figure 2.

(A) Dose-response curve of ESA for MDA-MB-468 cells. MDA-MB-468 cells were treated with ESA at the indicated concentrations for 24 h, and assayed for cell viability. Data shown are the mean ± SD (n = 3). LC50: 22 μM.

(B) MDA-MB-468 cells were pretreated with either iDGAT1/2 (10 μM), Triacsin C (5 μM), or Fer-1 (5 μM) for 0.5 h, treated with ESA (20 μM) for 24 h, and assayed for cell viability. Data shown are the mean ± SD (n = 3).

Hirata et al., Fig. S4.

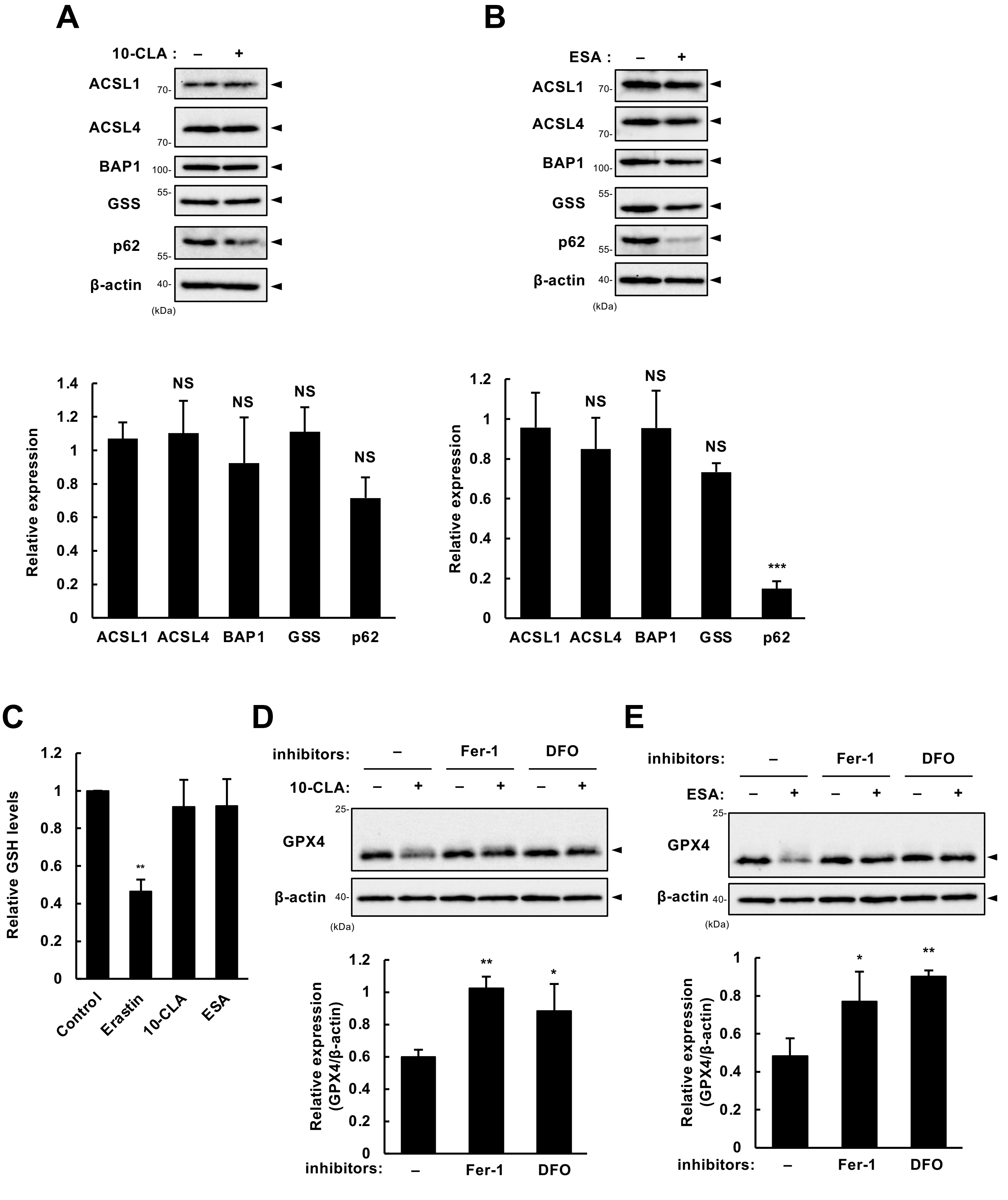

Supplementary Figure 5. CLA/CLNAs reduces GPX4 protein levels in a lipid peroxidation-dependent fashion without affecting GSH levels. Related to Figure 3.

(A, B) Immunoblot of lysates from HT1080 cells treated with 10-CLA (200 μM) or ESA (20 μM) for 5 h, using antibodies against the indicated proteins. Images are cropped for clarity; full-length blots are presented in Supplementary Fig. 13O-13P.

(C) Relative GSH levels of HT1080 cells treated with either 10-CLA (200 μM) or ESA (20 μM) or Erastin (40 μM) for 5 h. Data shown are the mean ± SD (n = 3).

(D, E) Immunoblot of lysates from HT1080 cells pretreated with Fer-1 (5 μM) or DFO (100 μM) and treated with 10-CLA (200 μM) (D) or ESA (20 μM) (E) for 5 h, using antibodies against the indicated proteins. Images are cropped for clarity; full-length blots are presented in Supplementary Fig. 13Q-13R.

Hirata et al., Fig. S5.

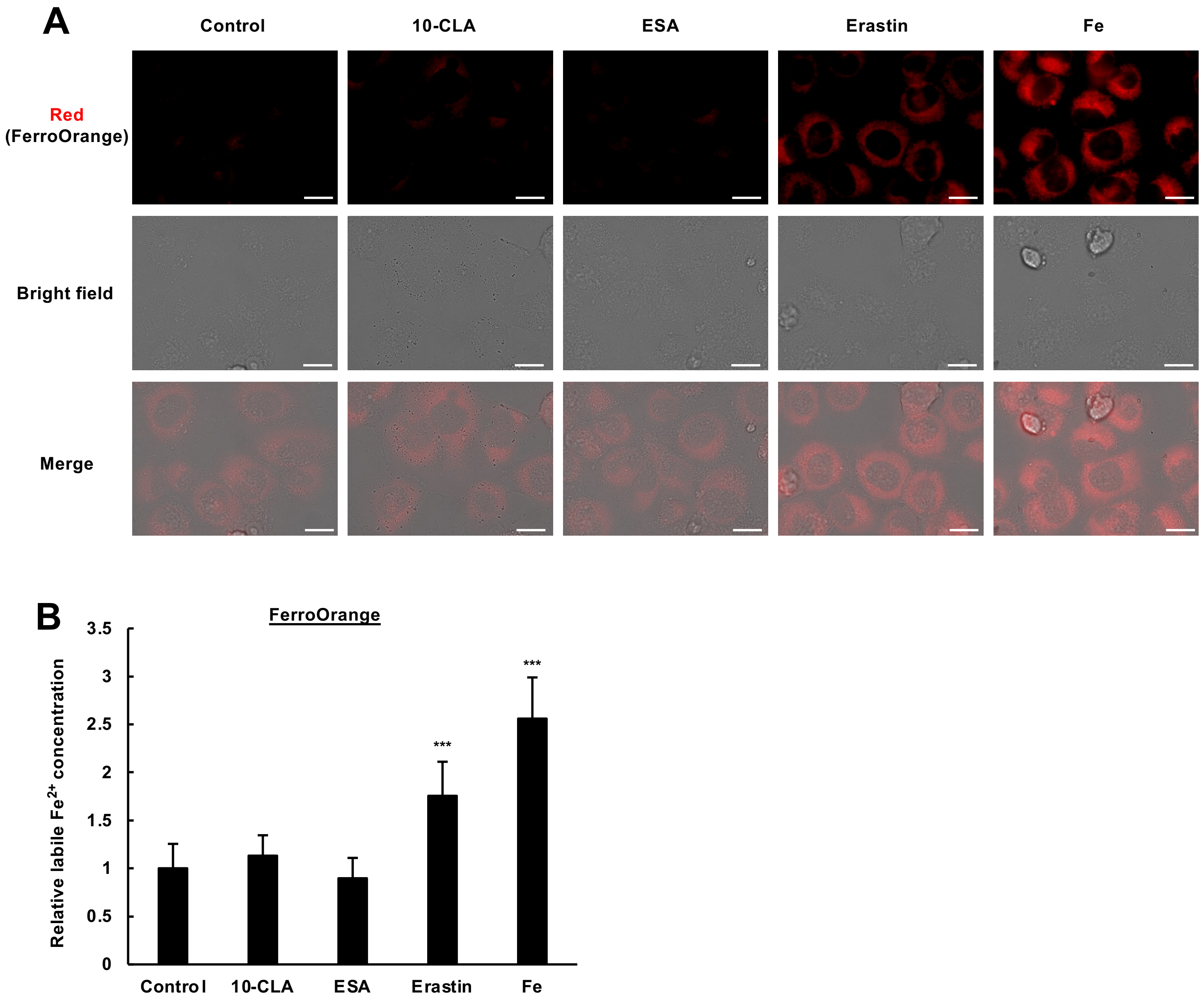

Supplementary Figure 6. CLA/CLNAs do not affect intracellular Fe^{2+} levels. Related to Figure 3.
(A, B) Fe^{2+} fluorescence staining in HT1080 cells using FerroOrange. Representative images of HT1080 cells treated with either 10-CLA (200 μM), ESA (20 μM), Erastin (20 μM) or Fe^{2+} (100 μM) for 4 h (A). Bar, 10 μm. Quantification of cellular red fluorescence intensity was performed in 3 fields for each sample, and shown as relative Fe^{2+} levels (mean ± SEM, n = 28 ~ 32) (B). ***p < 0.001 (vs control).

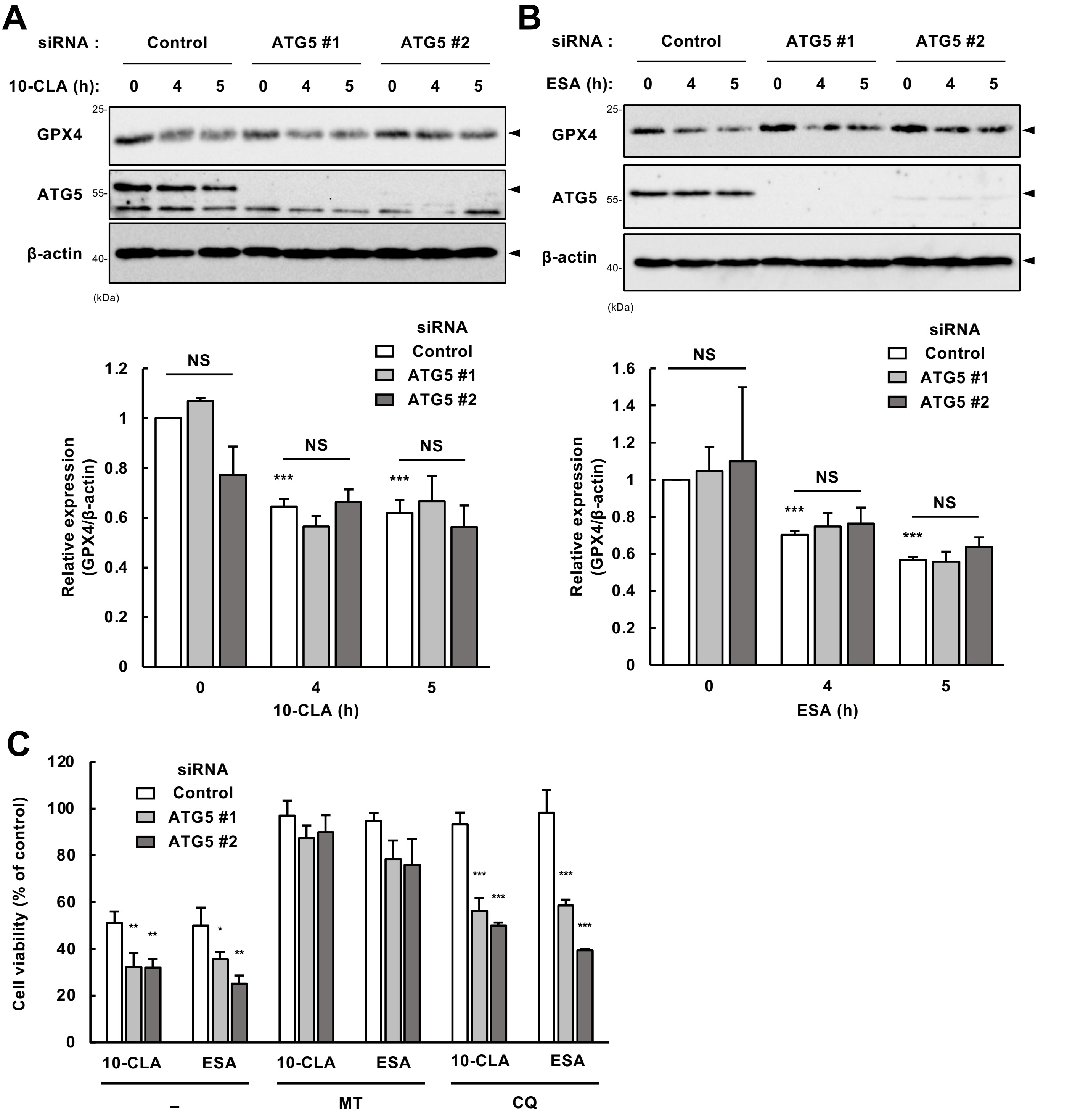

Supplementary Figure 7. *ATG5* knockdown does not recover GPX4 degradation and cell death induced by CLA/CLNAs. Related to Figure 4.

(A, B) Immunoblot of lysates from control- and *ATG5*-knockdown HT1080 cells treated with 10-CLA (200 μM) or ESA (20 μM) for indicated time, using antibodies against the indicated proteins. Below the blots, quantification of the band intensity of GPX4 normalized with that of β-actin was shown as the mean ± SEM (n = 3). Images are cropped for clarity; full-length blots are presented in Supplementary Fig. 13S-13T.

(C) Control- and *ATG5*-knockdown HT1080 cells were pretreated with MT (20 μM), CQ (20 μM) for 0.5 h, treated with 10-CLA (200 μM) or ESA (10 μM) for 24 h, and assayed for cell viability. Data shown are the mean ± SD (n = 3).

Hirata et al., Fig. S7.

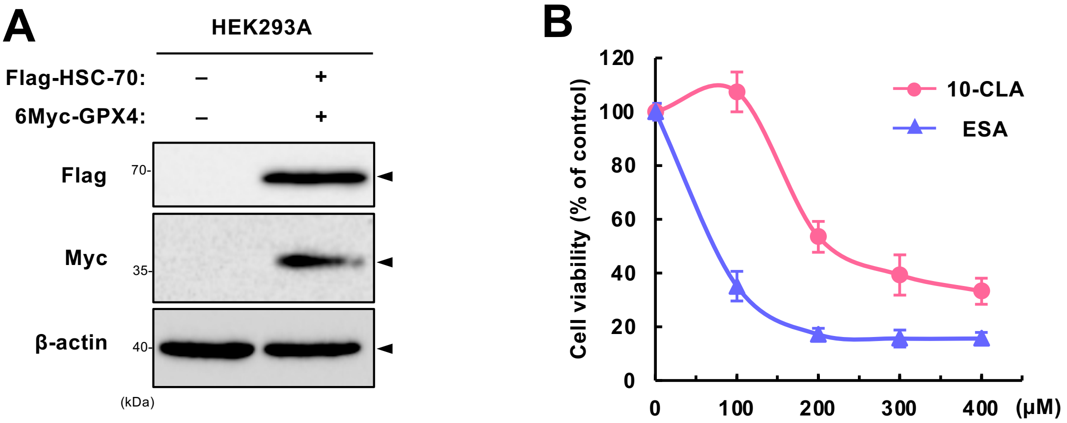

Supplementary Figure 8. Establishment of a stable HEK293A cell line expressing Flag-HSC70 and 6Myc-GPX4. Related to Figure 4.

(A) Immunoblot of lysates from HEK293A cells stably expressing Flag-HSC70 and 6Myc-GPX4, using antibodies against the indicated proteins. Images are cropped for clarity; full-length blots are presented in Supplementary Fig. 13U.

(B) HEK293A cells stably expressing Flag-HSC70 and 6Myc-GPX4 were treated with the indicated concentrations of 10-CLA or ESA for 24 h, and assayed for cell viability. Data shown are the mean ± SD (n = 3).

Hirata et al., Fig. S8.

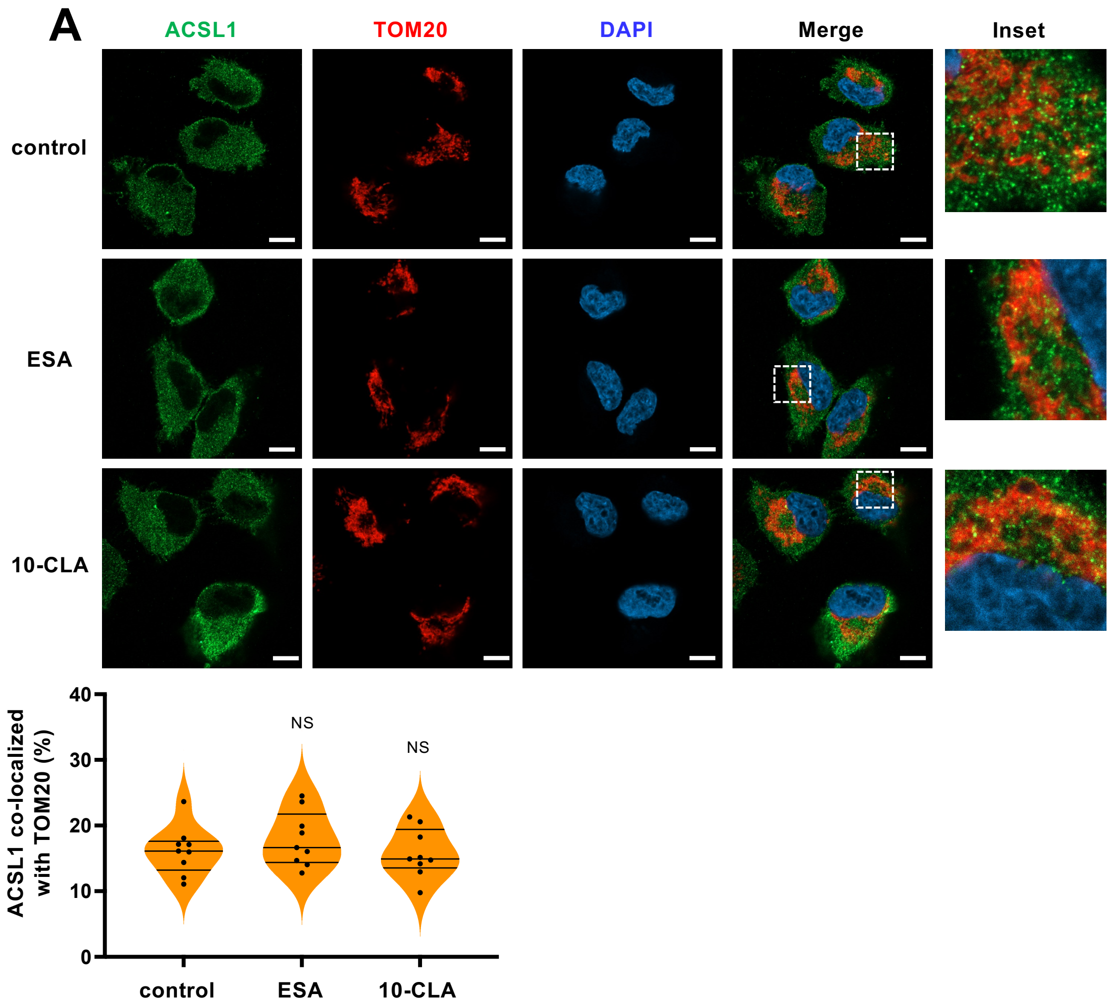

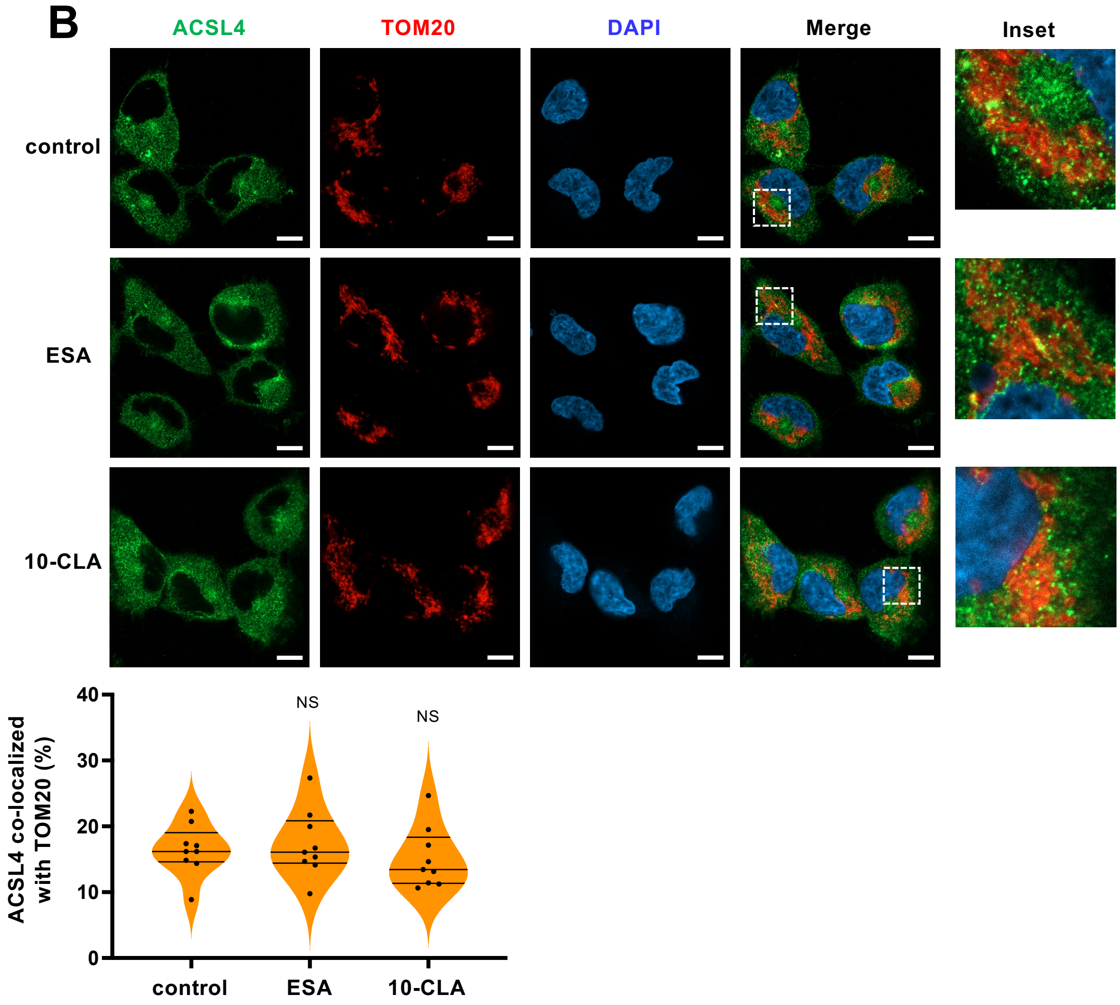

Hirata et al., Fig. S9.

C

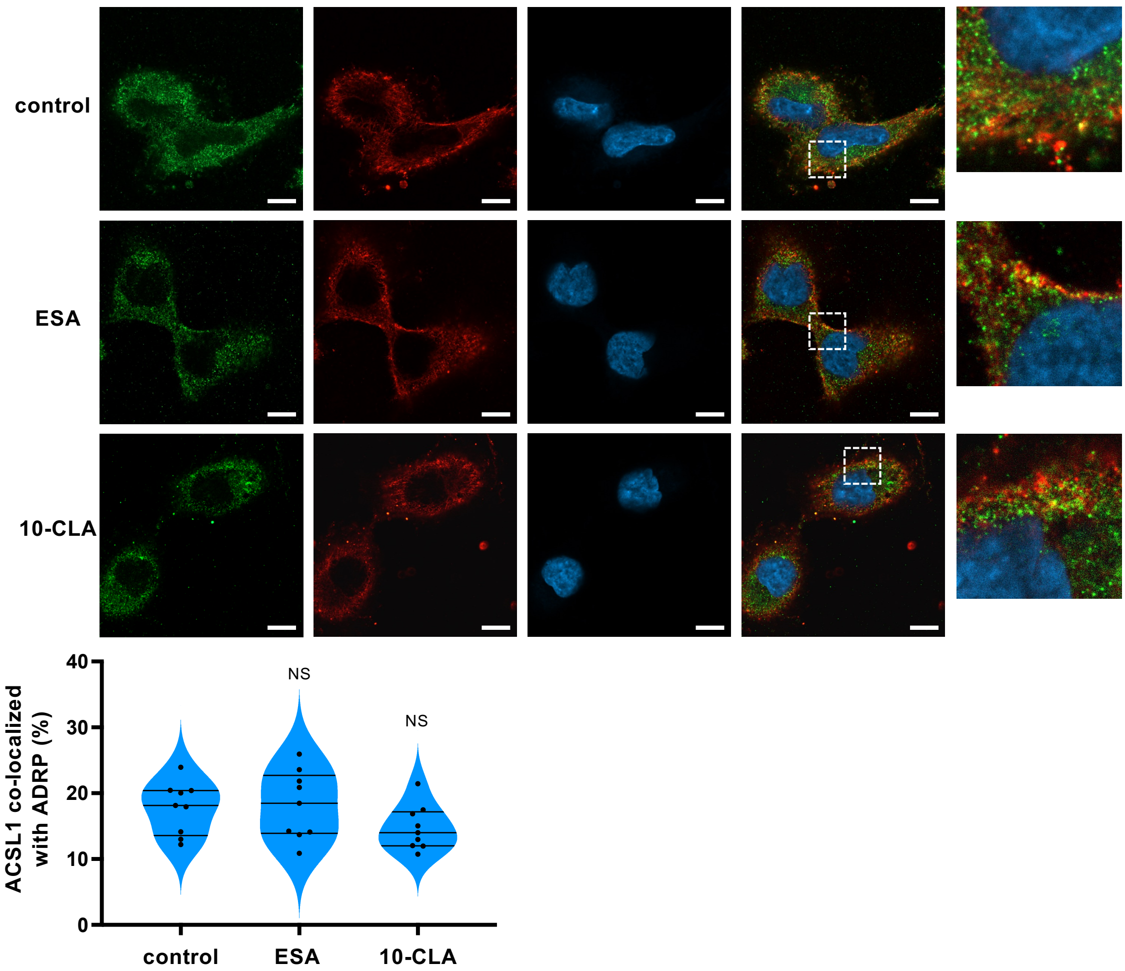

D

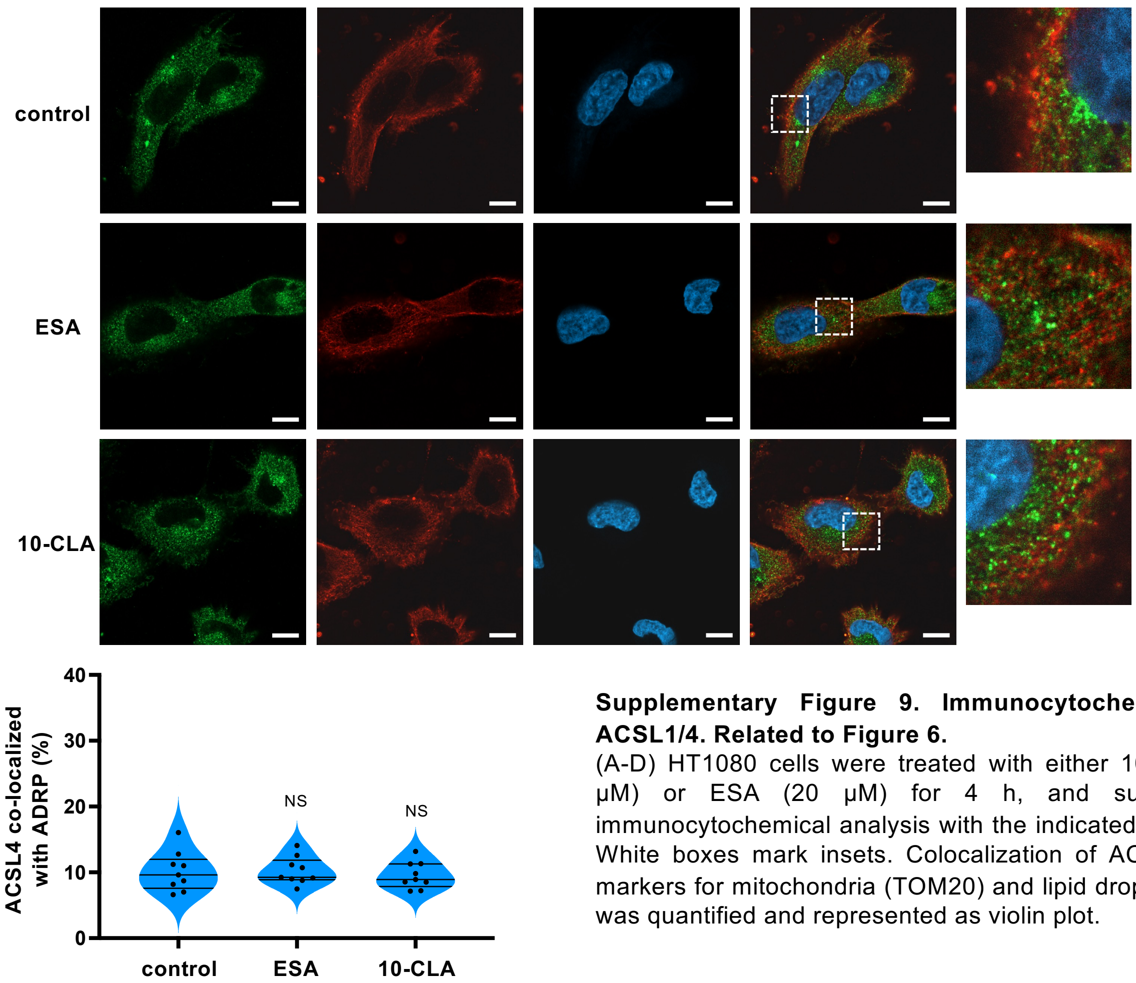

Supplementary Figure 9. Immunocytochemistry for ACSL1/4. Related to Figure 6.
(A-D) HT1080 cells were treated with either 10-CLA (200 μM) or ESA (20 μM) for 4 h, and subjected to immunocytochemical analysis with the indicated antibodies. White boxes mark insets. Colocalization of ACSL1/4 with markers for mitochondria (TOM20) and lipid droplet (ADRP) was quantified and represented as violin plot.

Hirata et al., Fig. S9.

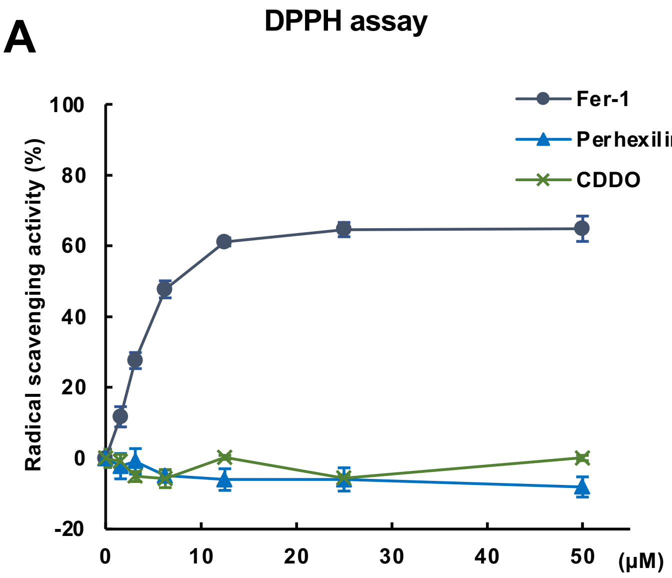

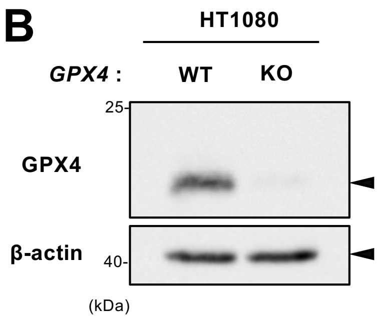

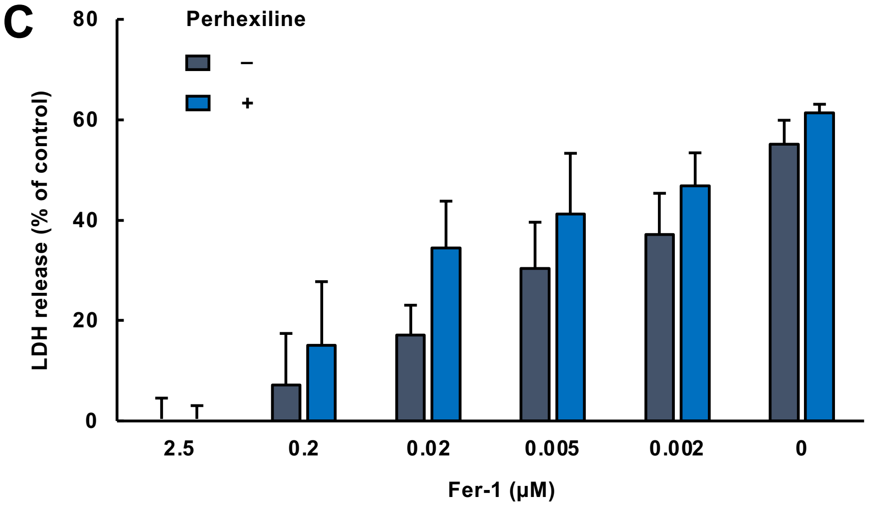

Supplementary Figure 10. Perhexiline and CDDO do not have an antioxidant activity. Related to Figure 6.
(A) The antioxidant activity of Perhexiline, CDDO, and Fer-1 was assayed using DPPH. (B) Immunoblot for GPX4 and β-actin in WT and *GPX4* KO HT1080 cells. Images are cropped for clarity; full-length blots are presented in Supplementary Fig. 13W.
(C) *GPX4* KO HT1080 cells maintained with 2.5 μM Fer-1 were seeded at 2500 cells on a 96-well plate in a medium with various concentrations of Fer-1 in the presence or absence of Perhexiline (7.5 μM). LDH release after 8 h was assessed and shown as the mean ± SD (n = 3).

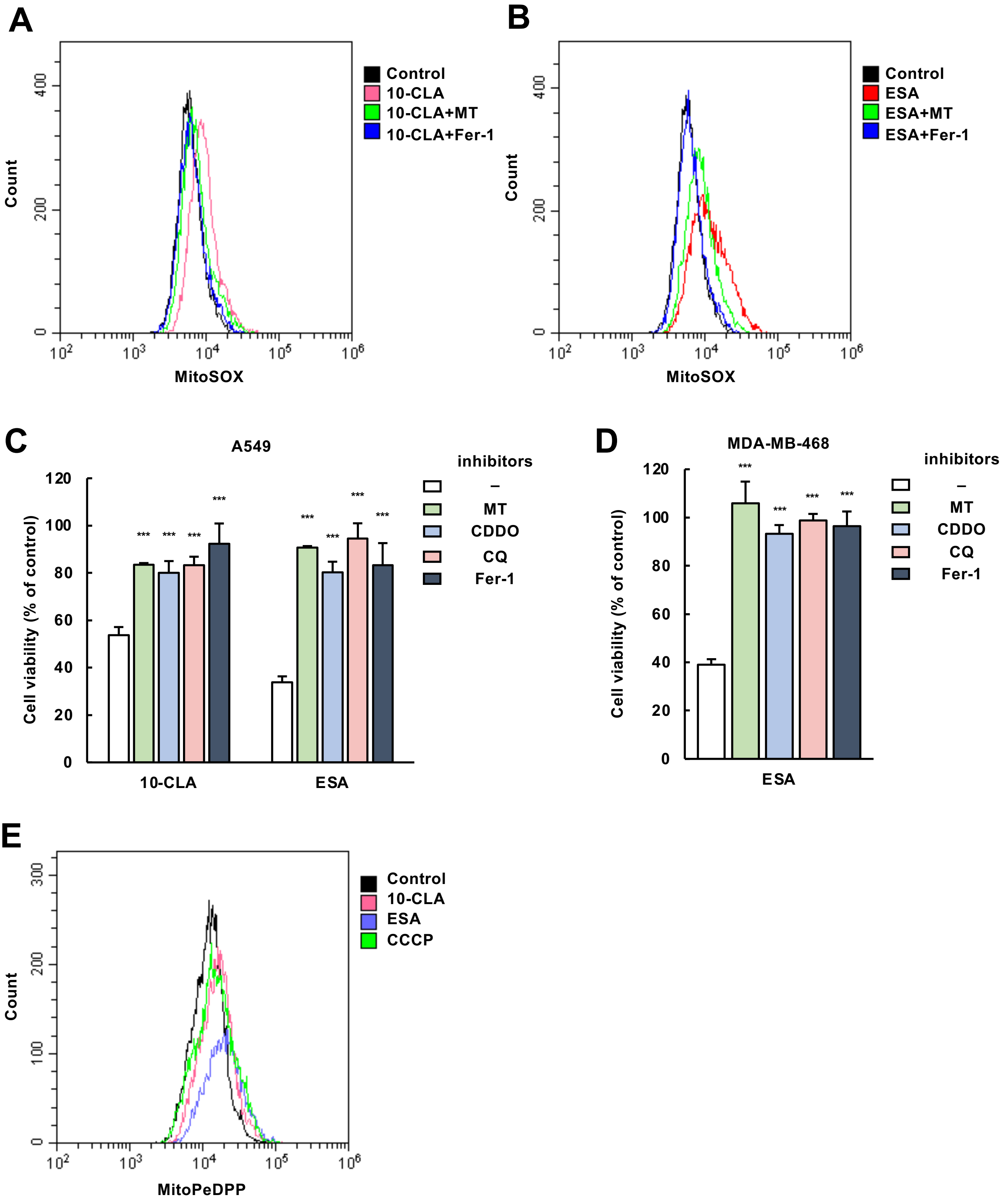

Supplementary Figure 11. CLA/CLNAs generate mitochondrial ROS/lipid ROS to promote ferroptosis. Related to Figure 6.

(A, B) Representative histograms of flow cytometric analysis for HT1080 cells treated with 10-CLA (200 μM) (A) and ESA (20 μM) (B) with or without MT (20 μM) and Fer-1 (5 μM) in Fig. 6A.

(C, D) A549 (C) and MDA-MB-468 (D) cells were pretreated with either MT (20 μM), CQ (20 μM), CDDO (10 μM in C, 5 μM in D), or Fer-1 (5 μM) for 0.5 h, treated with 10-CLA (200 μM) or ESA (20 μM) for 24 h, and assayed for cell. Data shown are the mean ± SD (n = 3).

(E) Representative histograms of flow cytometric analysis for HT1080 cells treated with either 10-CLA (200 μM), ESA (20 μM) or CCCP (10 μM) for 4 h detected with mitoPeDPP in Fig. 6I.

Hirata et al., Fig. S11.

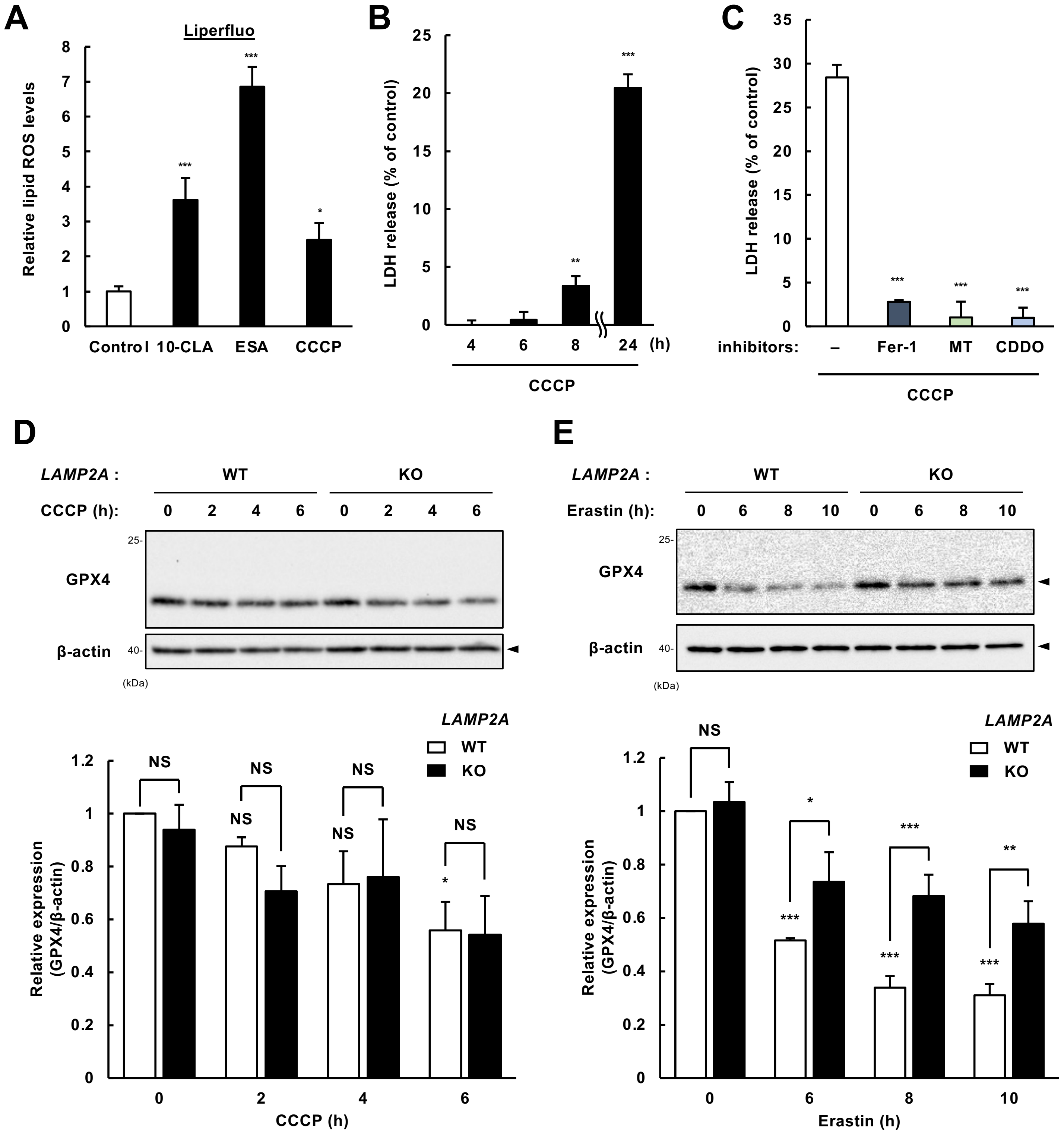

Supplementary Figure 12. CCCP triggers lipid ROS generation, GPX4 degradation and ferroptosis. Related to Figure 6.

(A) Lipid peroxidation levels of HT1080 cells treated with either 10-CLA (200 μM), ESA (20 μM) or CCCP (10 μM) for 4 h, detected with Liperfluo. Data shown are the mean ± SD (n = 3).

(B) HT1080 cells were treated with CCCP (10 μM) for indicated time, and subjected to cell death assay. Data shown are the mean ± SD (n = 3).

(C) HT1080 cells were pretreated with either Fer-1 (5 μM), MT (20 μM), or CDDO (5 μM) for 0.5 h, treated with CCCP (10 μM) for 24 h, and subjected to cell death assay. Data shown are the mean ± SD (n = 3).

(D, E) Immunoblot of lysates from WT and *LAMP2A* KO HT1080 cells treated with CCCP (10 μM) (D) or Erastin (20 μM) (E) for indicated time, using antibodies against GPX4 and β-actin.

Hirata et al., Fig. S12.

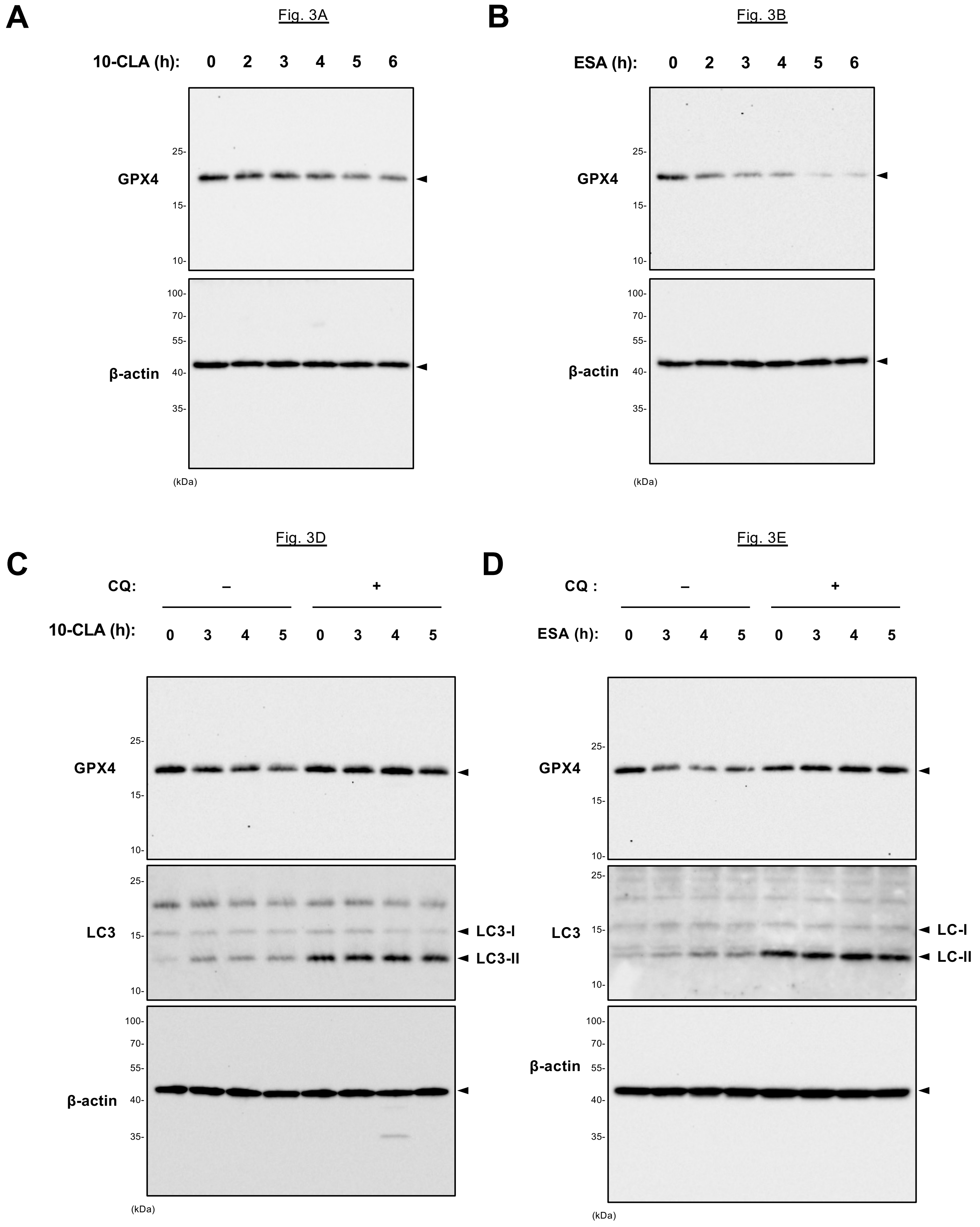

Hirata et al., Fig. S13.

E

Fig. 3F

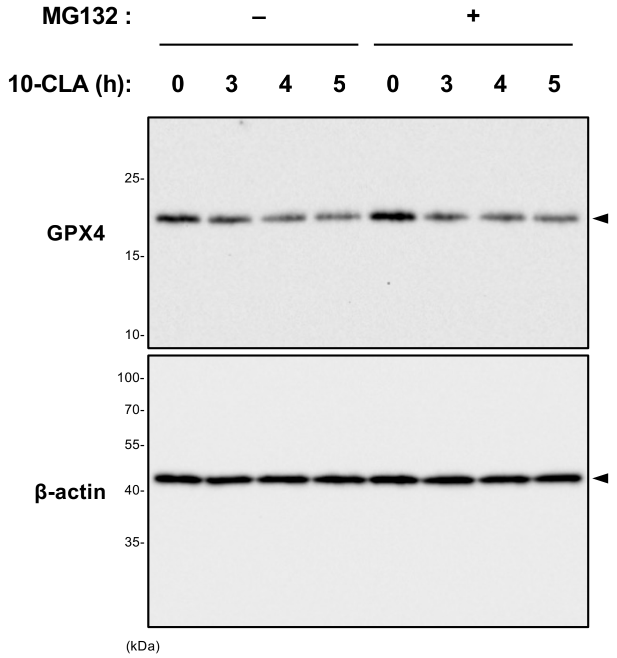

F

Fig. 3G

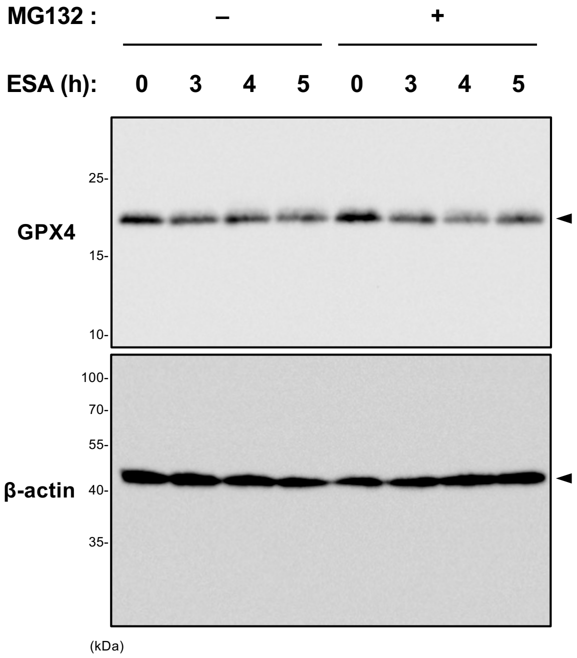

G

Fig. 4A

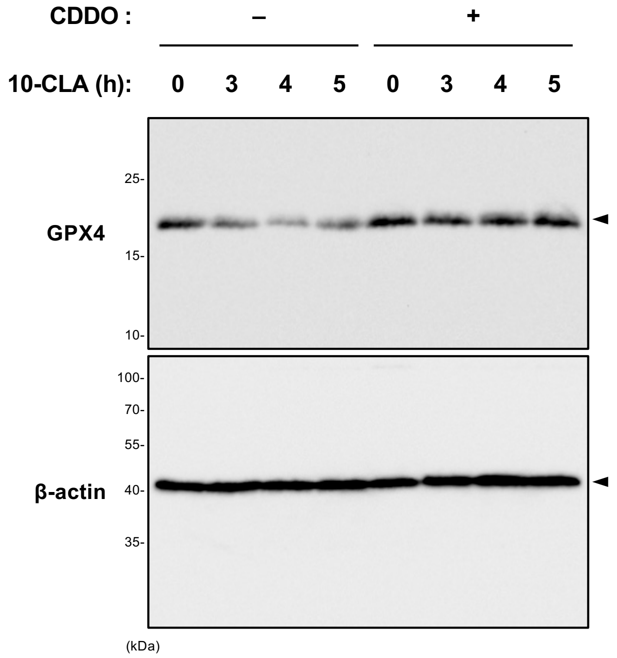

H

Fig.4B

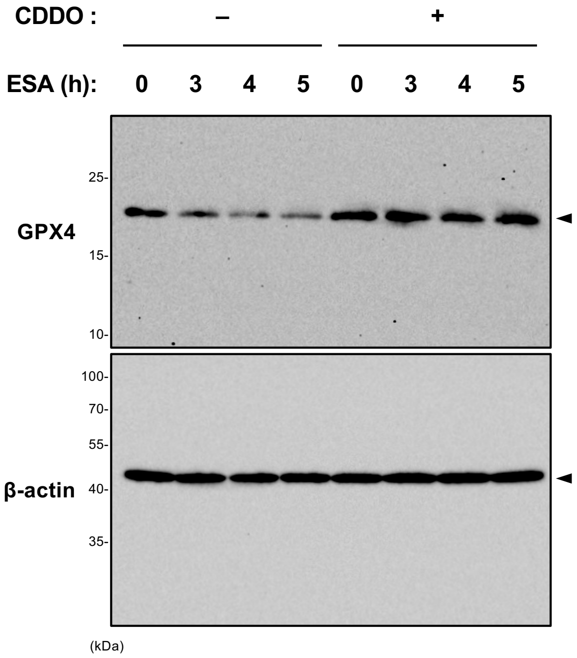

Hirata et al., Fig. S13.

I

Fig. 4D

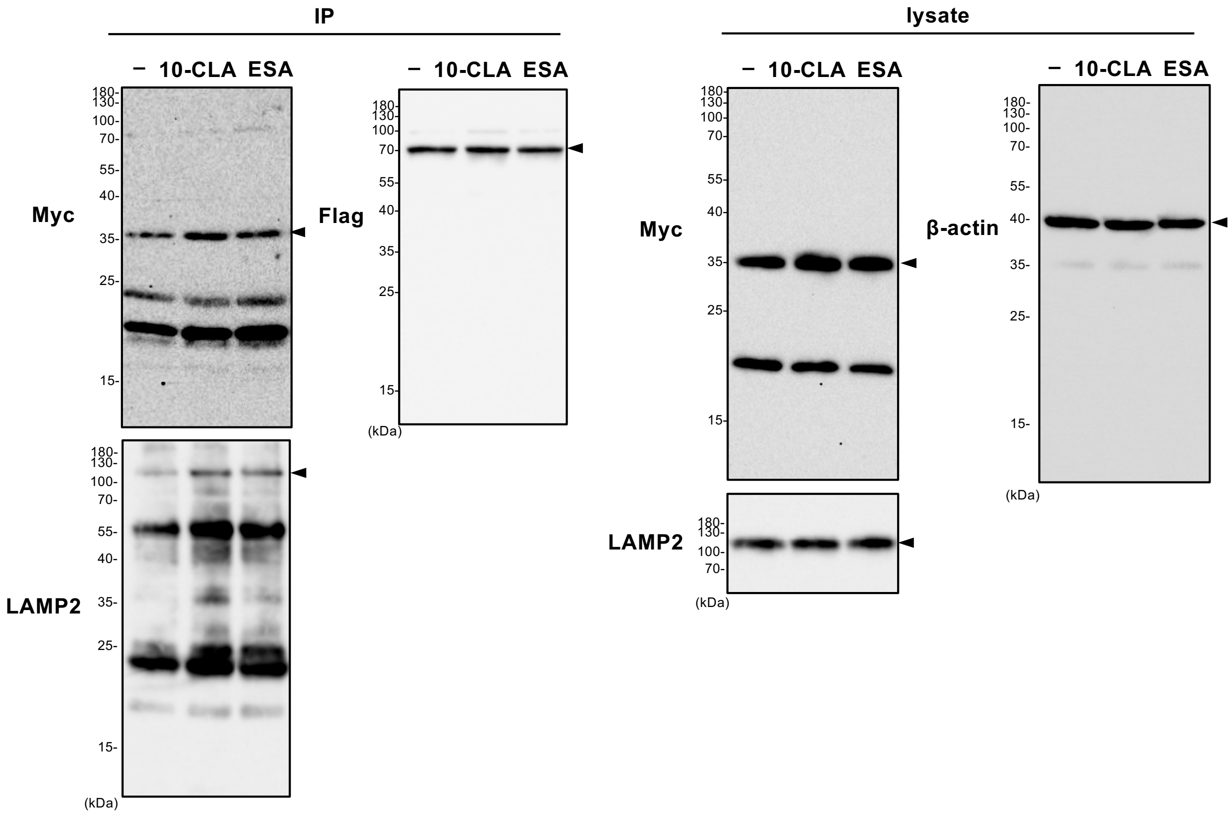

J

Fig. 5A

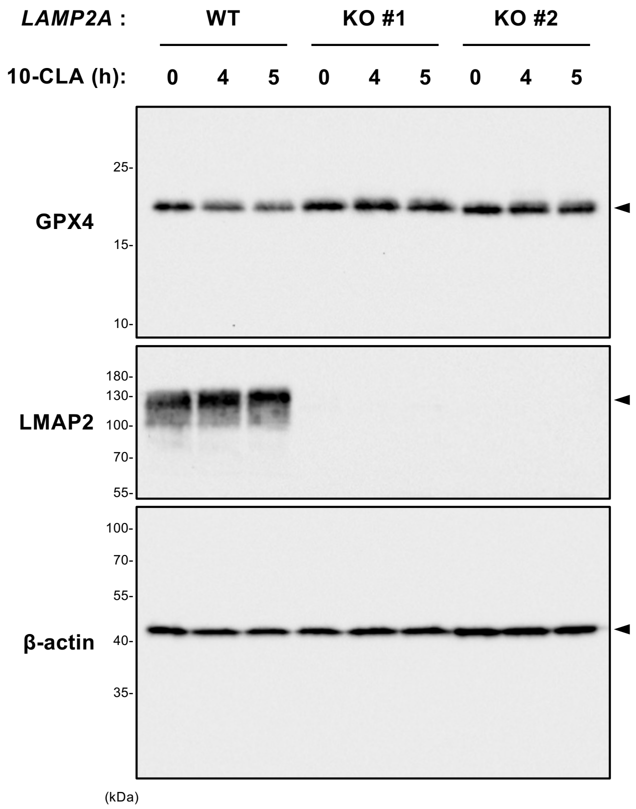

K

Fig. 5B

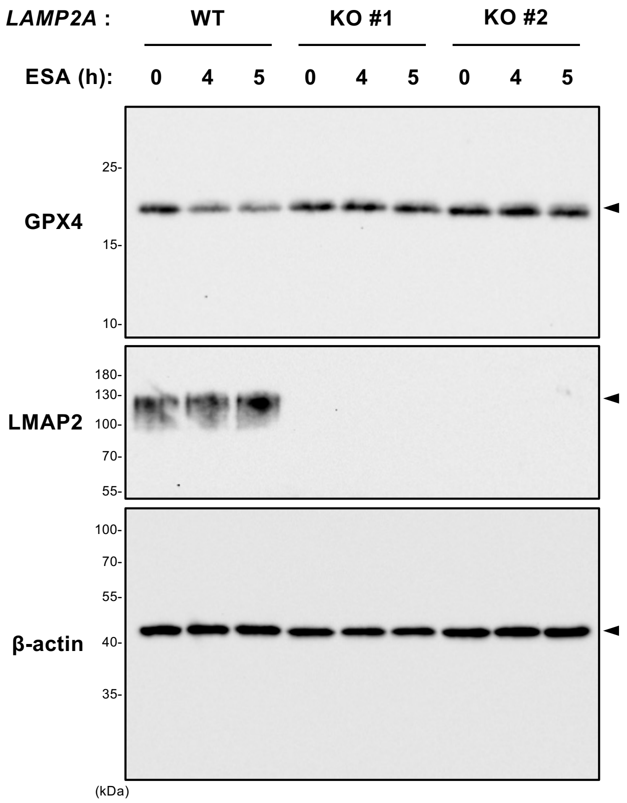

Hirata et al., Fig. S13.

L Fig. 6C

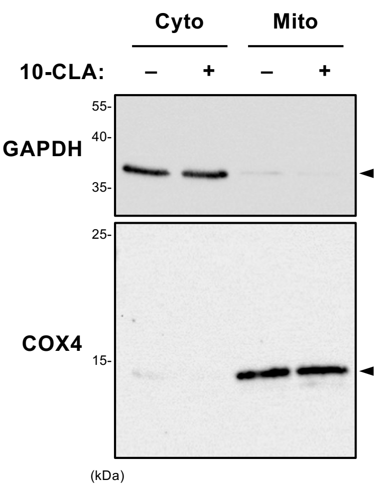

M Fig. 6G

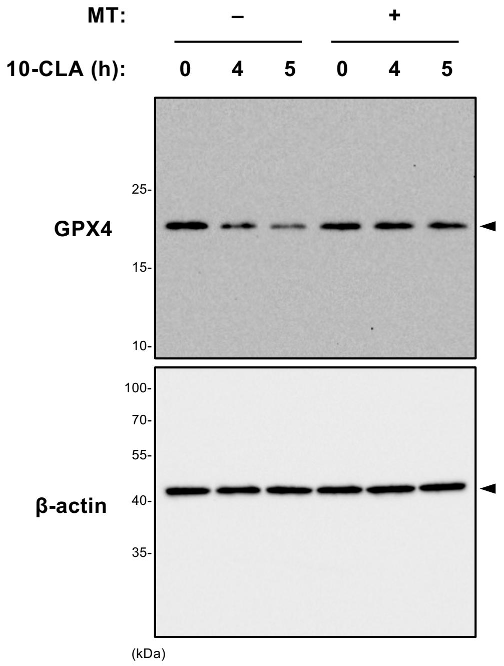

N Fig. 6H

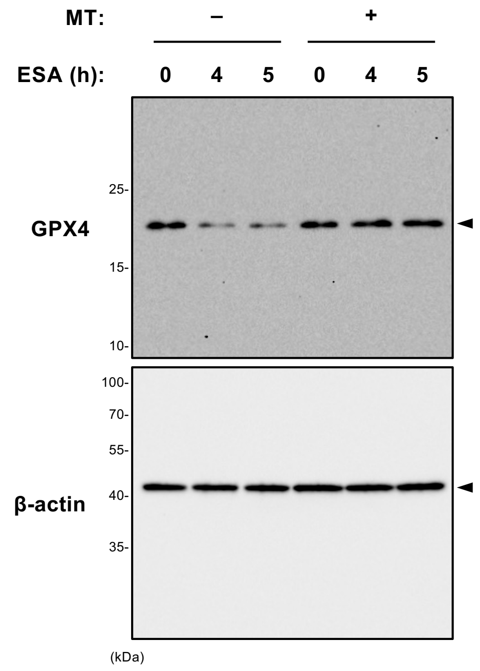

O Fig.S5A

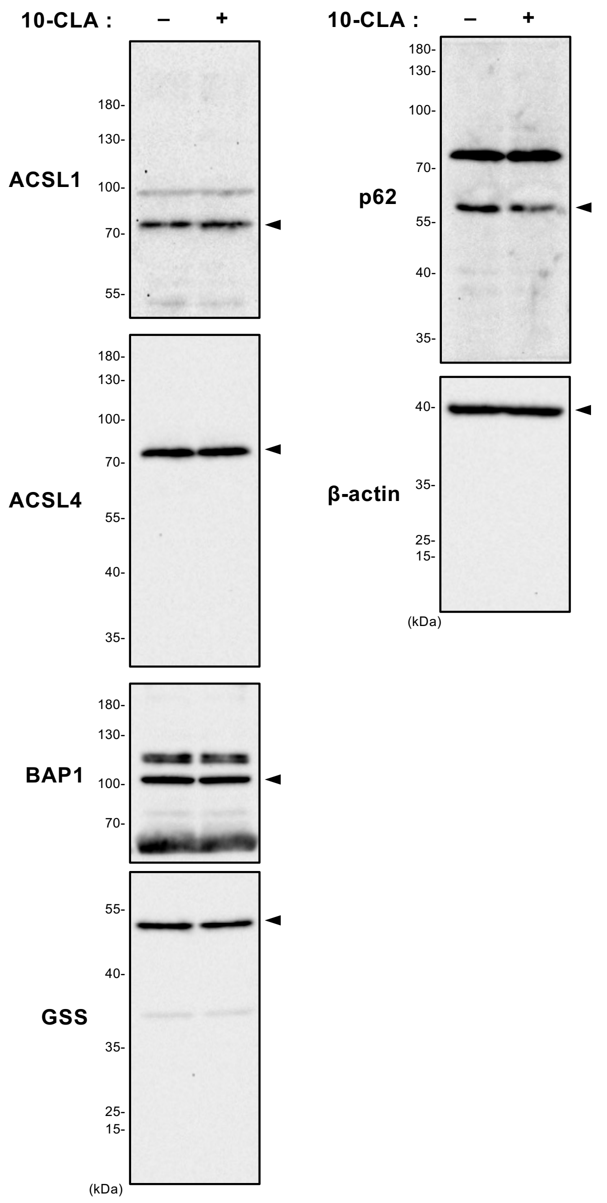

P Fig.S5B

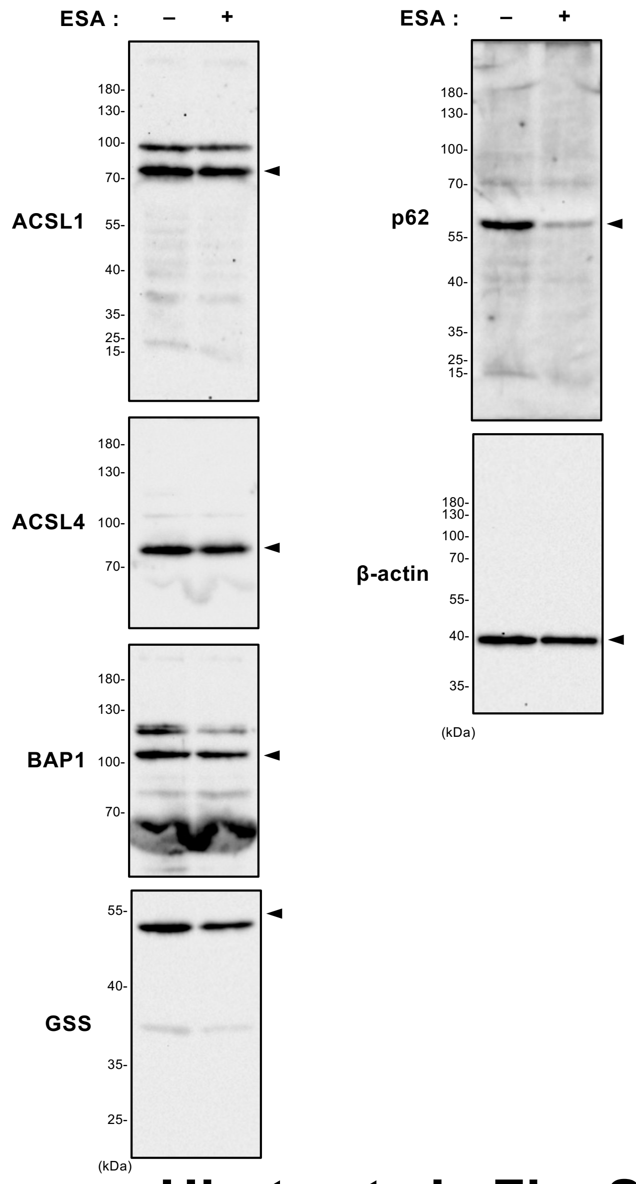

Hirata et al., Fig. S13.

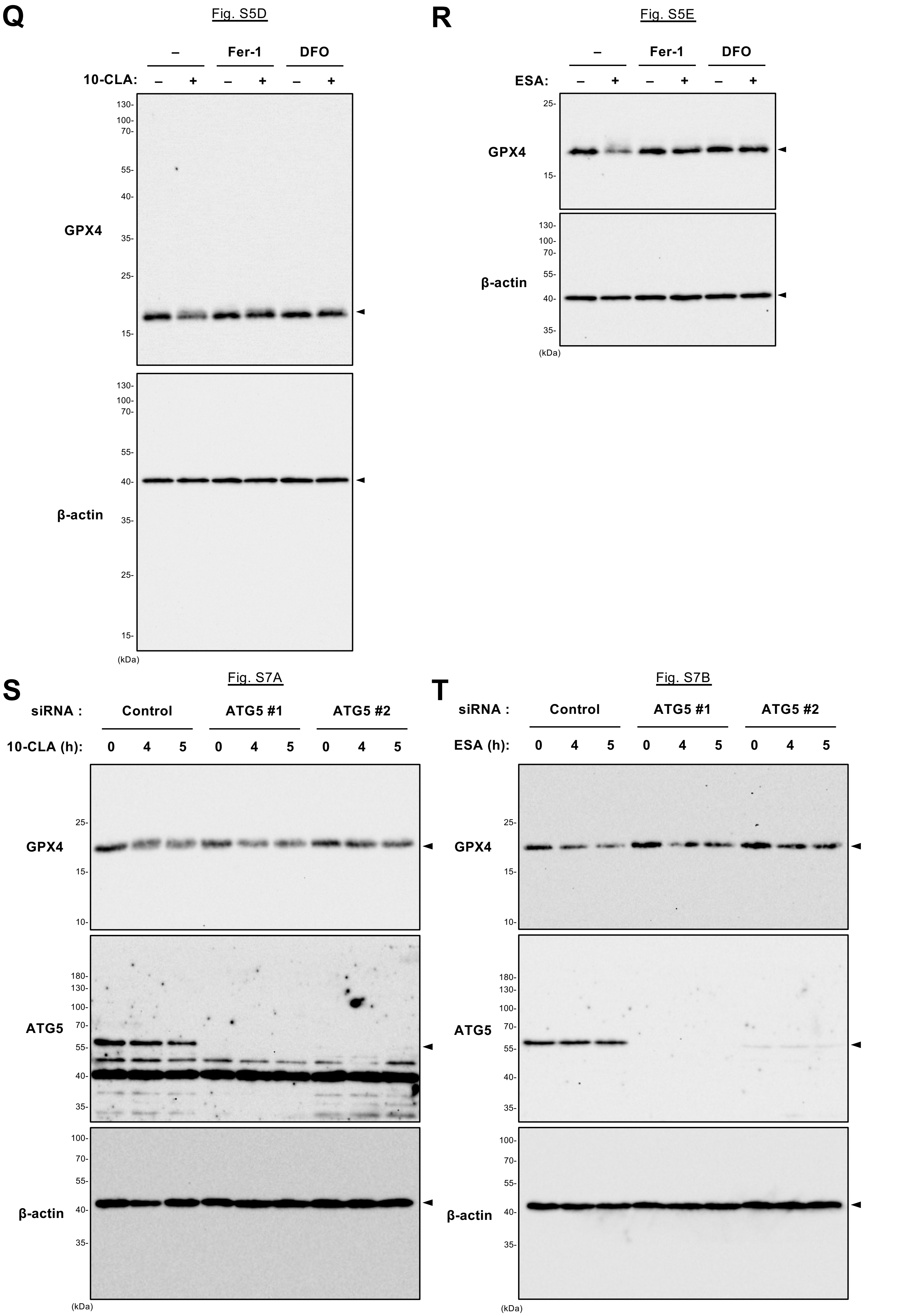

Hirata et al., Fig. S13.

U

Fig. S8A

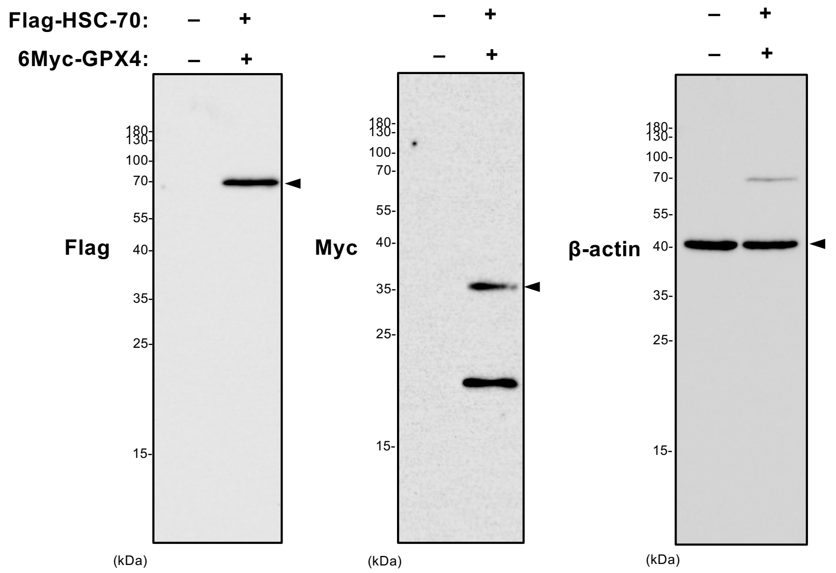

V

Fig. S10B

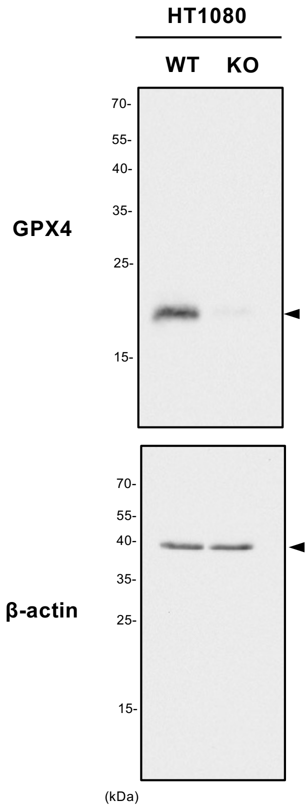

Supplementary Figure 13. Full scans of immunoblot data.

(A-V) Uncropped images of Fig. 3A (A), Fig. 3B (B), Fig. 3D (C), Fig. 3E (D), Fig. 3F (E), Fig.3G (F), Fig. 4A (G), Fig. 4B (H), Fig. 4D (I), Fig. 5A (J), Fig. 5B (K), Fig. 6C (L), Fig. 6G (M), Fig. 6H (N), Fig. S5A (O), Fig. S5B (P), Fig. S5D (Q), Fig. S5E (R), Fig. S7A (S), Fig. S7B (T), Fig. S8A (U), and Fig. S10B (V).

Hirata et al., Fig. S13.
